# In vitro and in vivo inhibition of malaria parasite infection by monoclonal antibodies against *Plasmodium falciparum* circumsporozoite protein (CSP)

**DOI:** 10.1038/s41598-021-84622-x

**Published:** 2021-03-05

**Authors:** Merricka C. Livingstone, Alexis A. Bitzer, Alish Giri, Kun Luo, Rajeshwer S. Sankhala, Misook Choe, Xiaoyan Zou, S. Moses Dennison, Yuanzhang Li, William Washington, Viseth Ngauy, Georgia D. Tomaras, M. Gordon Joyce, Adrian H. Batchelor, Sheetij Dutta

**Affiliations:** 1 Structural Vaccinology Lab, Malaria Biologics Branch, Walter Reed Army Institute of Research, Silver Spring, MD USA; 2 Malaria Biologics Branch, Walter Reed Army Institute of Research, Silver Spring, MD USA; 3 US Military HIV Research Program, Walter Reed Army Institute of Research, Silver Spring, MD USA; 4 The Henry M. Jackson Foundation for the Advancement of Military Medicine, Bethesda, MD USA; 5 Malaria Department, Naval Medical Research Center, Silver Spring, MD USA; 6 Center for Human Systems Immunology, Duke University Medical Center, Durham, NC USA; 7 Departments of Surgery, Duke University Medical Center, Durham, NC USA; 8 Departments of Immunology, Duke University Medical Center, Durham, NC USA; 9 Departments of Molecular Genetics and Microbiology, Duke University Medical Center, Durham, NC USA; 10 Duke Human Vaccine Institute, Duke University Medical Center, Durham, NC USA; 11 Statistics and Epidemiology Branch, Walter Reed Army Institute of Research, Silver Spring, MD USA

**Keywords:** Immunology, Microbiology, Structural biology, Diseases, Molecular medicine

## Abstract

*Plasmodium falciparum* malaria contributes to a significant global disease burden. Circumsporozoite protein (CSP), the most abundant sporozoite stage antigen, is a prime vaccine candidate. Inhibitory monoclonal antibodies (mAbs) against CSP map to either a short junctional sequence or the central (NPNA)_n_ repeat region. We compared in vitro and in vivo activities of six CSP-specific mAbs derived from human recipients of a recombinant CSP vaccine RTS,S/AS01 (mAbs 317 and 311); an irradiated whole sporozoite vaccine PfSPZ (mAbs CIS43 and MGG4); or individuals exposed to malaria (mAbs 580 and 663). RTS,S mAb 317 that specifically binds the (NPNA)_n_ epitope, had the highest affinity and it elicited the best sterile protection in mice. The most potent inhibitor of sporozoite invasion in vitro was mAb CIS43 which shows dual-specific binding to the junctional sequence and (NPNA)_n_. In vivo mouse protection was associated with the mAb reactivity to the NANPx6 peptide, the in vitro inhibition of sporozoite invasion activity, and kinetic parameters measured using intact mAbs or their Fab fragments. Buried surface area between mAb and its target epitope was also associated with in vivo protection. Association and disconnects between in vitro and in vivo readouts has important implications for the design and down-selection of the next generation of CSP based interventions.

## Introduction

According to the WHO, 228 million cases and 405,000 deaths were caused by malaria in 2018^1^. Eradication is a top priority for many national governments and international organizations as insecticide and prophylactic drugs have been unable to stop mortality due to malaria. The most abundant protein of the invasive sporozoite stage of the malaria parasite is the circumsporozoite protein (CSP), and antibodies to CSP can block hepatocyte infection by the sporozoite^2^. The N-terminal region of *Plasmodium falciparum* (Pf) CSP contains a putative proteolytic cleavage site and an inter-species conserved ‘Region I’^3^. The central portion comprises 4 amino-acid repeats consisting of a junctional sequence (NP-DPNA-NPNV-DPNA) and 25–42 (NPNA)_n_ or (NANP)_n_ major repeats interspersed with NPNV, DPNA minor repeats. While the tetrameric major and minor repeats are relatively conserved across all *P. falciparum* strains, the C-terminus of CSP is polymorphic and contains a cysteine-rich thrombospondin type-I repeat domain^4,5^. The C-terminal domain has been shown to directly interact with hepatocytes, and this interaction may require a proteolytic event in region I, but the role of CSP remains poorly understood^6^.

The most advanced malaria vaccine Mosquirix (GlaxoSmithKline) contains RTS,S antigen that is composed of (NANP)_19_ and the C-terminal domain of CSP fused to a Hepatitis B particle^7^, formulated with AS01 adjuvant. RTS,S vaccine can elicit > 80% protection against controlled malaria human infection (CHMI), but efficacy against natural infection is less than 50%^8,9^. A pediatric formulation of RTS,S/AS01 is currently undergoing Phase 4 trials at several field sites across Africa^10^. Immunization via mosquito bite with radiation-attenuated sporozoites (IMRAS vaccine)^11^, cryopreserved irradiated sporozoites (PfSPZ vaccine)^12^ or sporozoites delivered under chloroquine prophylaxis (PfSPZ-C vaccine)^13^ also elicit protection against CHMI that is in part mediated by inhibitory CSP antibodies. Highly protective polyclonal and monoclonal antibodies have been mapped to the central repeats and junctional sequence, but not to the N- or C-terminal regions of CSP^2,14–16^.

CSP monoclonal antibodies (mAbs) may be useful in short-term malaria prevention among high-risk populations like travelers, pregnant women, and infants^17^. CSP mAbs elicited by RTS,S (e.g. mAbs 317 and 311); PfSPZ (e.g. mAbs MGG4 and CIS43), PfSPZ-C (e.g. mAbs 4493 and 1210) or by natural exposure (e.g. mAbs 663 and 580) have been structurally and functionally characterized^12,18–22^. RTS,S-elicited mAb 317 reacts only to the (NPNA)_n_ repeats but the most potent mAbs elicited by whole sporozoites, e.g. mAbs CIS43 and MGG4, were less epitope specific; binding with high affinity to junctional and (NPNA)_n_ sequences^12,21^. Such antibodies termed as cross-binders by Murugan et al*.* appear to be resulting from affinity maturation of epitope-specific mAbs^22^. The repeat region is flexible but forms a series of discrete partially ordered motifs including type I β-turns^23^ or ‘3_10_ turns’^24^, and several CSP mAbs revealed homotypic contacts between adjacent Fabs allowing multiple Fabs to pack head-to-head or adjacent to each other along the CSP repeats^20,24^.

**Figure 1:**
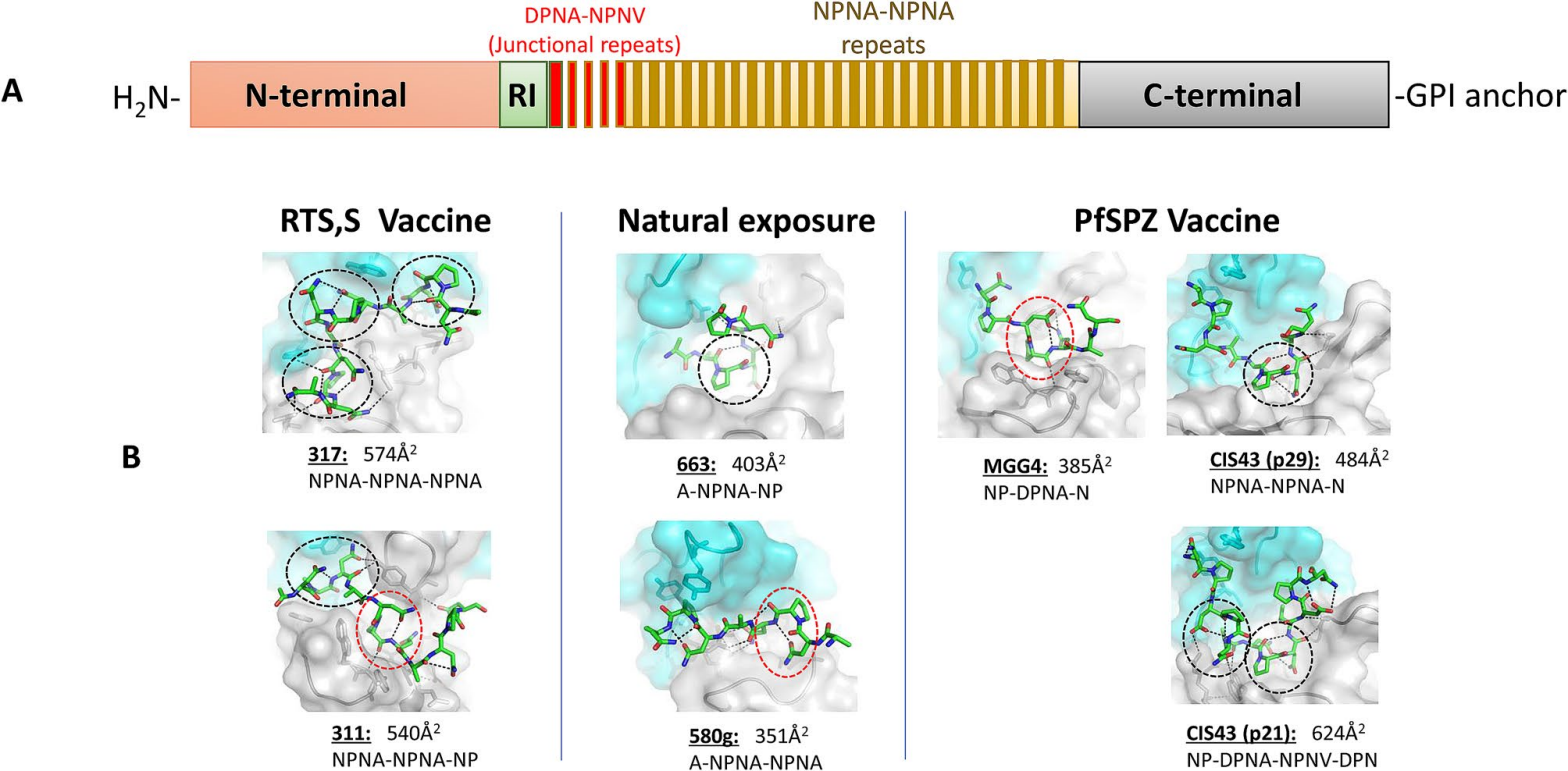
Structural analysis of mAb binding to protective *P. falciparum* Circumsporozoite protein (CSP) epitopes. (**a**) Schematic representation of full-length CSP (not to scale). (**b**) Antibody paratope structures depicting monoclonal antibody binding to representative repeat or junctional peptides: 311 (PDB code 6AXK), 317 (6AXL)^18^, CIS43 bound to peptide 21 (6B5M) and peptide 29 (6B5O)^12^, germline 580 (6AZM) and 663 (5BK0)^19^, MGG4 (6BQB)^21^. The contact area between the peptides and the complementarity determining regions, or the buried surface area (BSA), shown under each structure were calculated using the AREAIMOL program in the CCP4 software suite^43^. Heavy and light chains are colored blue and light grey, respectively. Peptides are depicted in stick conformations (green carbons). Hydrogen bonds are shown as black dashes. Type I β and 3_10_ turns are indicated by black and red circles, respectively.

Anti-CSP antibodies have relatively low number of somatic hypermutations (SHM) and are predominantly encoded by the *IGHV3* gene family^2,18,25^. Despite the structural heterogeneity of CSP repeats and the diverse manner in which they are recognized (Fig. 1), certain common features of CSP protective epitopes that bind to monoclonal antibodies with a wide range of affinities have emerged (Table S1). For many antibodies, the core NPNA motif is in a ‘3_10_ turn’ to which heavy chain tryptophan 52 sitting adjacent to the kinked proline of the peptide binds, as is seen in mAb 311 and 1210^20,24^. The tryptophan 52 was encoded by IGV3-33, the most commonly elicited heavy chain variable gene^22,25^. Similarly, a core DPNA motif forming a 3_10_ turn sits at the center of the mAb MGG4 binding site, and in this case tryptophan 52 was encoded by the related IGHV3-30 variable gene^21^. The core NPNA motif in a type I turn conformation sits at the center of the mAb 663 antigen binding site, and binds tyrosine 32 sitting adjacent to the peptide proline encoded by the IGHV3-23 gene^19^. The mAb 580 is encoded by IGHV3-23 gene, that makes use of light chain tyrosines to interact with the kinked proline^19^, such that mAb 580 has a lower buried surface area (BSA) and forms a lower affinity complex. For less common high affinity mAbs with a larger peptide recognition surface, rather than there being a core NPNA motif, two or three motifs are recognized. For example, mAb 317 recognizes three successive NPNA type I turns in an extensive interaction that involves many heavy as well as light chain contacts^18^. Mab CIS43 preferentially recognizes the junctional sequence and has DPNA NPNV containing two type I β turns at the center of the peptide-mAb complex^12^. Although, dual-specific mAb CIS43 preferentially recognizes the junctional sequence DPNA NPNV, it also binds to NPNA NPNA albeit with ~ tenfold lower affinity^12^.

Sporozoites delivered under the skin through the bite of an infected mosquito reach the liver where they traverse through hepatic tissue before invading a hepatocyte. Traversal inhibition assays^26–28^, exoerythrocytic stage development inhibition assays^19,29,30^ and humanized mouse liver parasite burden inhibition assays^31^ have been used to dissect the mechanism of anti-CSP activity. Tan et al*.* showed that mAbs like MGG4 that recognized the junctional sequence were potent neutralizers^21^ and Kisalu et al*.* showed high level inhibition of hepatocyte invasion and protection by the dual-specific mAb CIS43^12^. Murugan et al*.* screened a panel of such cross-binders (including mAbs 4493 and CIS43) along with epitope-specific mAbs (including mAb 317) to demonstrate that high affinity to (NANP)_n_ determined the traversal inhibition activity and there was no significant difference in the in vivo protective response of high affinity cross-binding, dual-specific and epitope-specific mAbs against transgenic parasite challenge^22^.

We functionally characterized a panel of repeat region mAbs to enable rational vaccine and immune-prophylactic development. MAbs 317, 311, CIS43, MGG4, 580 and 663 were compared head-to-head by direct ELISA (quantity, binding strength and specificity), avidity ELISA (binding strength), biolayer interferometry assay (kinetic parameters and affinity), in vitro inhibition of liver stage development assay (ILSDA), in vivo protection (transgenic parasite challenge) and the sporozoite reaction assay (precipitation of CSP on live sporozoites). MAb 317 emerged as most protective in vivo but mAb CIS43 was a better inhibitor of sporozoite invasion into human hepatocytes in vitro. Overall, our data strongly supports the continued evaluation of CSP mAbs as immuno-prophylactics.

## Results

### Epitope-specificity

**Figure 2:**
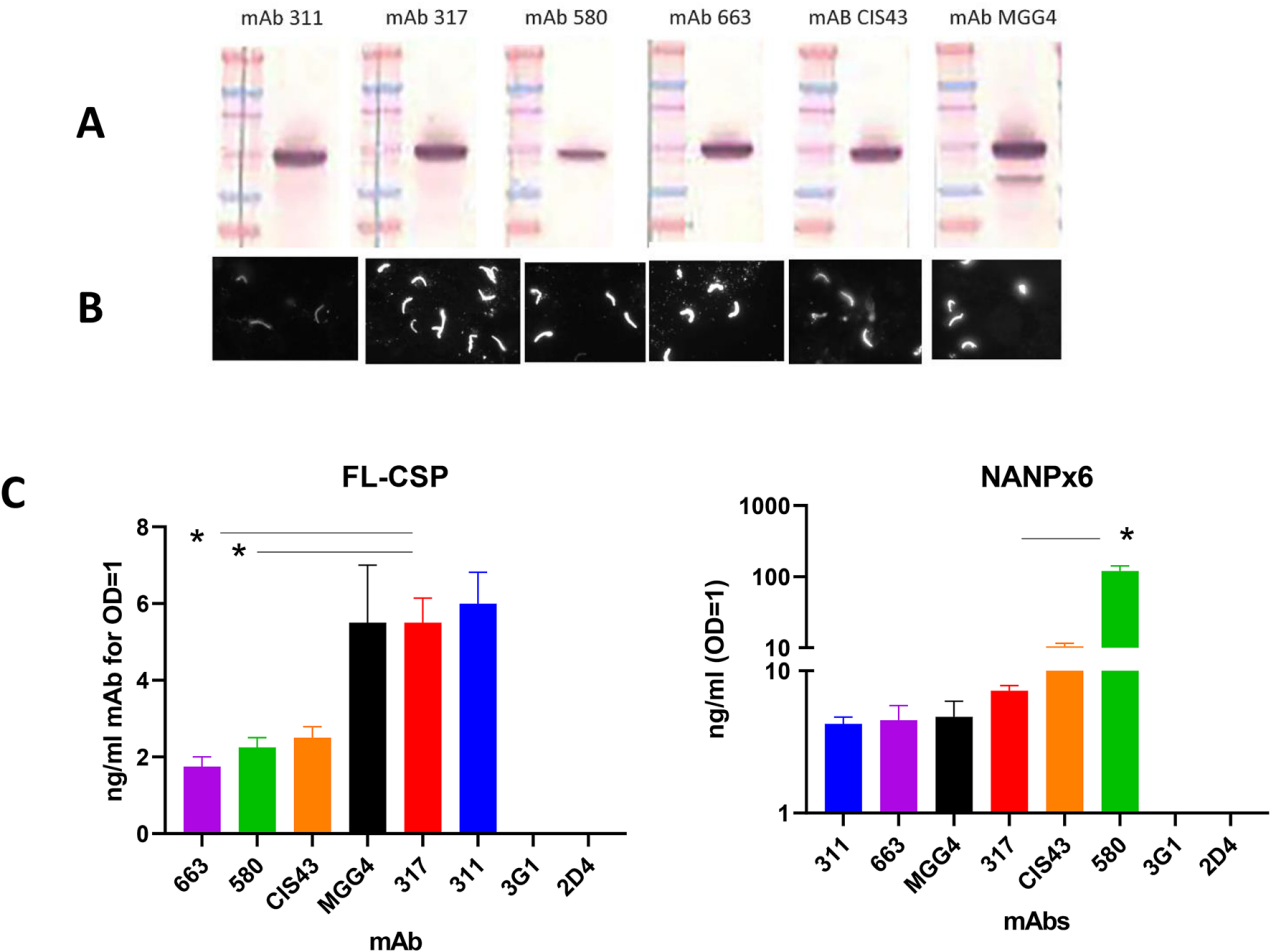
Immuno-reactivity of mAbs. (**A**) Western blot, (**B**) Immunofluorescence assay against *Plasmodium falciparum* NF54 strain sporozoites. (**C**) ELISA reactivity (ng/ml mAb needed for OD = 1) of mAbs against FL-CSP protein and NANPx6 peptide. Mean and standard error from four independent experiments; mAbs 2D4 and 3G1 were negative controls. Comparisons against mAb 317; (*) indicate level of significance for ng/ml mAb differences determined by ANOVA and *p* values corrected for multiple comparisons by Tukey’s method.

Using information from protein data bank (PDB) files, Fab 311, 317, CIS43, MGG4, 663, and 580 sequences were grafted onto a common IgG1 Fc sequence and full-length mAbs were expressed in mammalian cells^12,18,19,21^. Purified recombinant mAbs (Fig. S1) demonstrated reactivity with *P. falciparum* sporozoite CSP by western blot (Fig. 2A) and immuno-fluorescence assay (Fig. 2B). Binding capacity of the mAbs (ng/ml needed for OD = 1) was compared in a direct ELISA. All mAbs efficiently bound to a nearly full-length recombinant FL-CSP (< 6 ng/ml). Compared to the RTS,S mAb 317, two natural infection mAbs 663, 580 bound more efficiently (*p* < 0.05) (Fig. 2C). Binding to the short NANPx6 peptide was also highly efficient (< 10 ng/ml), except for mAb 580. The RTS,S mAb 317 bound more efficiently to NANPx6 than mAb 580 (*p* < 0.05) (Fig. 2C).

**Figure 3:**
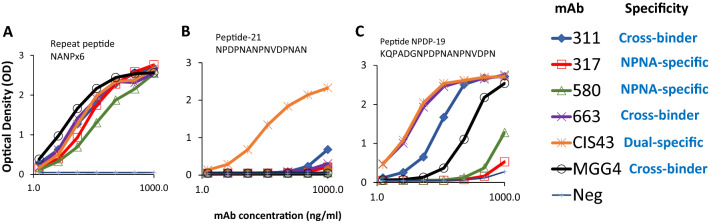
Epitope-specificity. Raw ELISA curves showing mAb binding patterns: (**A**) Target peptide NANPx6, (**B**) peptide-21 (NPDPNANPNVDPNAN) and (**C**) peptide NPDP-19 (KQPADGNPDPNANPNVDPN. Negative control wells (Neg) contained no primary antibody; a representative of three independent ELISA experiments was plotted.

To classify binding specificities, a peptide ELISA was conducted comparing binding to the repeat peptide NANPx6, peptide-21 (NP-DPNA-NPNV-DPNA-N)^12^, and peptide NPDP-19 (KQPADGNP-DPNA-NPNV-DPN)^21^ (Fig. 3). MAbs 317 and 580 were confirmed as NPNA-specific (binding NANPx6 but not peptide 21 or NPDP_19_)^18,19,32^. MAbs MGG4, 663, and 311 bound NANPx6 and NPDP-19 but not peptide 21, and were categorized as “cross-binders” according to Murugan et al*.*^22^. MAbs CIS43 bound all three peptides confirming that it was truly “dual-specific” for the junctional and (NPNA)_n_ sequence as described by Kisalu et al*.*^12^.

### Affinity by biolayer interferometry (BLI)

Affinities of mAbs within this panel were reported previously against a variety of peptide and protein targets (Table S1). To directly compare their affinities head-to-head, the monovalent Fabs and bivalent mAbs were tested by a BLI-based assay to determine the association rate constant (*k*_a_), dissociation rate constant (*k*_*d*_) and equilibrium dissociation constant (*K*_D_) against the NANPx6 peptide and CSP antigen (raw data: Figs. S2, S3, Table S2).

**Figure 4:**
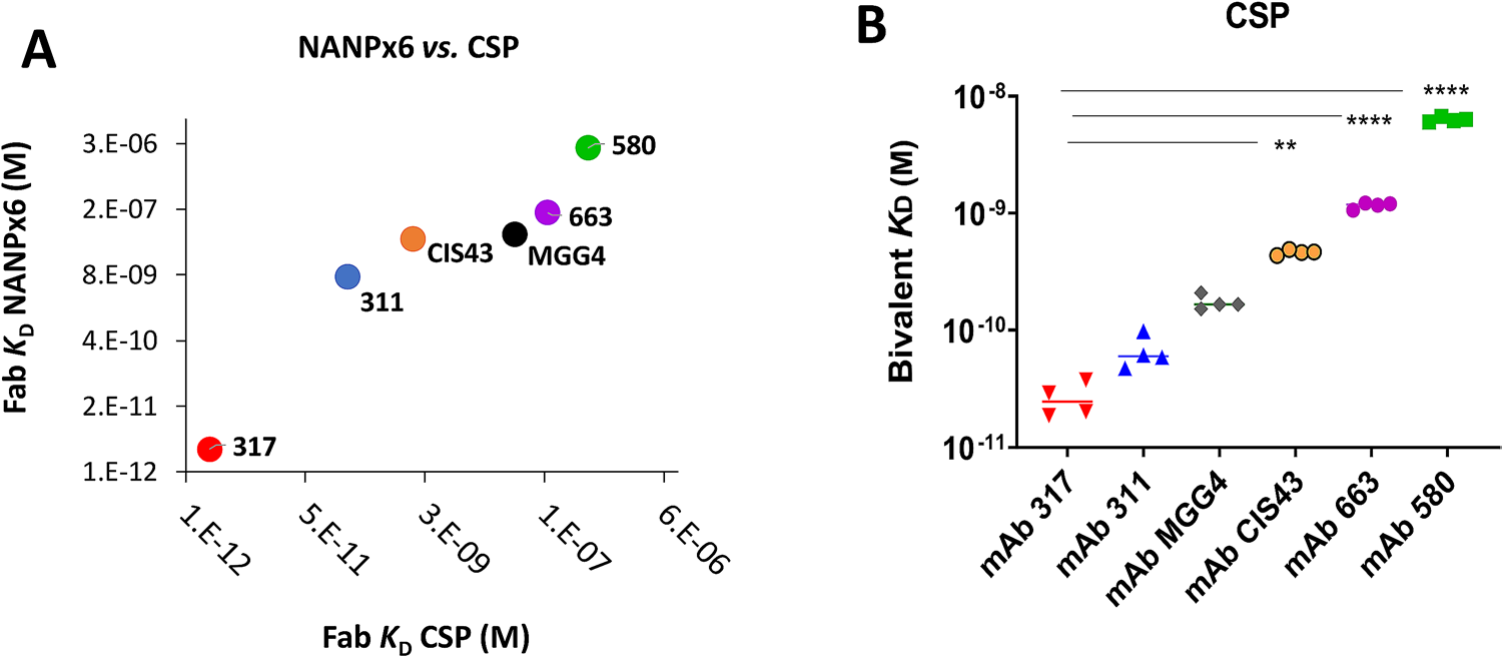
Affinity by Biolayer Interferometry (BLI). (**A**) Antibody binding fragment (Fab) dissociation constant (*K*_D_) against NANPx6 *vs.* CSP antigen determined by BLI (Octet; ForteBio, Freemont CA). Global fit to a 1:1 Langmuir binding model was used, experiment was performed twice on at least four concentrations of Fab (raw data Table S2, Figs. S2, S3). (**B**) Mean bivalent mAb dissociation constants (*K*_D_) against FL-CSP determined by BLI performed as four independent experiments on two different Octet instruments **(**raw data Table S2). Comparisons against mAb 317; (*) indicate level of significance for *K*_D_ value differences determined by ANOVA and *p* values corrected for multiple comparisons by Tukey’s method.

Rate constants *k*_a_ (10^4^ to 10^5^ M^−1^ s^−1^) and *k*_*d*_ values (10^–1^ to 10^–7^ s^−1^) yielded wide ranging *K*_D_ values for Fab fragments binding to the NANPx6 antigen (Table 1). The highest affinity to NANPx6 peptide was obtained for Fab 317 with the slowest dissociation rate (*k*_*d*_ = 1.7 × 10^–7^ s^−1^) and lowest dissociation constant (*K*_D_ = 3.0 pM). The lowest affinity to NANPx6 was obtained for Fab 580, with the fastest dissociation rate and the highest dissociation constant (*K*_D_ = 2.6 μM) (Fig. 4A; y*-axis)*. The affinity of the dual-specific Fab CIS43 for NANPx6 (*K*_D_ = 41 nM) was four orders of magnitude lower than the NPNA-specific Fab 317 (Table 1). A parallel BLI analysis against the nearly full-length FL-CSP antigen also confirmed the highest affinity for Fab 317 (*K*_D_ = 2 pM); 800-fold higher than Fab CIS43 and 2 × 10^5^-fold higher than Fab 580 (Fig. 4A; *x-axis*). For Fab fragments, the ratios of *K*_D_ values against NANP/CSP were ≥ 1. In particular, for the cross-reactive Fabs CIS43 and 311, the binding affinities to CSP were 24 and 36-fold higher than to NANPx6, respectively (Table 1). Pearson correlation analysis (Table S3) showed a positive association between Fab affinity to NANPx6 and to the FL-CSP antigen (*r* = 0.98; *p* = 0.001), suggesting (NPNA)_n_ was likely a preferred binding epitope on FL-CSP.

**Table 1:** Kinetic analysis of mAbs using bio-layer interferometry (BLI).

mAb	Fab binding NANPx6 target antigen			Fab binding FL-CSP antigen			Bivalent mAb binding FL-CSP target antigen			Ratio of Fab *K*_D_ NANPx6/CSP	Ratio of *K*_D_ Fab/mAb
	*K*_D_ (nM)	*k*_*a*_ (M^−1^ s^−1^)	*k*_*d*_ (s^−1^)	*K*_D_ (nM)	*k*_*a*_ (M^−1^ s^−1^)	*k*_*d*_ (s^−1^)	*K*_D_ (nM)	*k*_*a*_ (M^−1^ s^−1^)	*k*_*d*_ (s^−1^)		
311	7	2.7E+04	2.0E−04	0.2	1.0E+05	2.1E−05	0.07	4.8E+05	3.07E−05	35.8	3
317	0.003	5.9E+04	1.7E−07	0.002	1.2E+05	2.6E−07	0.03	1.1E+06	2.97E−05	1.3	0.08
CIS43	41	4.0E+04	1.7E−03	2	2.8E+04	4.8E−05	0.46	1.5E+06	6.92E−04	24.4	4
MGG4	51	9.0E+04	4.5E−03	47	5.1E+04	2.4E−03	0.17	4.7E+05	8.16E−05	1.1	275
663	138	4.0E+05	5.6E−02	139	1.3E+05	1.8E−02	1	9.7E+05	1.12E−03	1.0	120
580	2650	1.5E+05	3.9E−01	524	1.9E+05	9.7E−02	6	2.1E+06	1.29E−02	5.1	83

Equilibrium dissociation constant (*K*_D_), association rate constant (*k*_a_), and dissociation rate constant (*k*_d_) of the antigen-binding fragment (Fab) and intact monoclonal antibodies (mAbs). Ratios represent *K*_D_ values of Fab binding to NANPx6 / FL-CSP antigen; and ratio of *K*_D_ values of Fab / intact mAbs binding to FL-CSP.

Bivalent (intact) mAb affinities were also determined against the FL-CSP antigen^33^ (Table 1). Association rate constant *k*_a_ for the bivalent mAbs were similar (10^5^–10^6^ M^−1^ s^−1^) and approached the diffusion limit for biological molecules^34^. In-line with the Fab data, intact mAb 317 affinity (*K*_D_ = 30 pM) was the highest and the difference between mAb 317 affinity and that of mAbs CIS43, 663 and 580 were statistically significant (all *p* values < 0.05) (Fig. 4B). Pearson correlation analysis for bivalent versus Fab affinities to CSP showed a positive association (*r* = 0.99; *p* < 0.001) (Table S3). Remarkably, bivalent forms of three low affinity mAbs 580, 663 and MGG4 bound to CSP with 83 to 275-fold higher affinity than their respective Fab fragments, whereas conversely, the highest affinity Fab 317 bound to CSP with 12-fold higher affinity than its bivalent form (Table 1). This suggested that Fabs 580, 663 and MGG4 may cooperate to stabilize their peptide interactions, whereas 317 binds more efficiently as an isolated Fab.

### Avidity by ELISA

**Figure 5:**
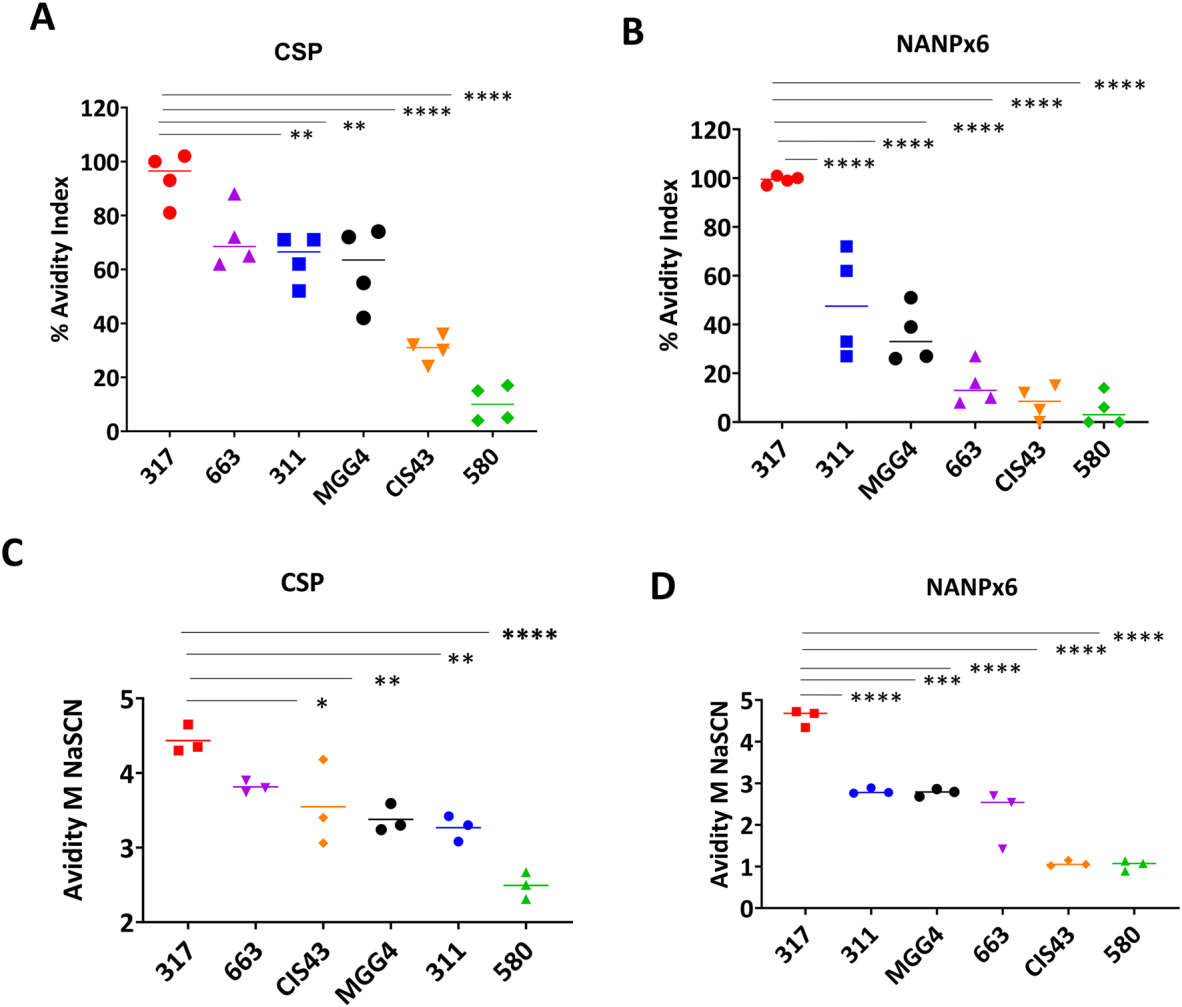
Avidity index (AI). AI for the CSP mAbs determined using 2 M Sodium Thiocyanate (NaSCN) wash method against (**A**) FL-CSP antigen or (**B**) NANPx6 antigen. Avidity was also determined using washing with a serial dilution of NaSCN against (**C**) FL-CSP antigen or (**D**) NANPx6 plate antigen. Mean data and individual values from three or more independent experiments arranged from highest to lowest. Comparisons against mAb 317; (*) indicate level of significance for mean differences estimated by ANOVA and *p* values corrected for multiple comparisons by Tukey’s method.

The term avidity refers to the average strength of vaccine-induced polyclonal antibody binding to a target antigen^8,35–38^. In an ELISA, the proportion of mAb that remained bound to CSP or NANPx6 (avidity index; AI), after a 2 M sodium thiocyanate (NaSCN) wash, was estimated on a 0–100% scale^39^ (Fig. 5A, B). In an alternative ELISA methodology, 0–6 M NaSCN wash solutions were used, and the molarity of NaSCN that reduced optical density by half-maximal provided an estimate of avidity (Fig. 5C, D). Average avidity determined by these two methods against both CSP and NANPx6 antigens showed a positive association (Pearson’s coefficient *r* = 0.8 and 0.9; *p* < 0.0001; Fig. S4A, B; Table S3).

The two avidity assay methodologies confirmed BLI data against both FL-CSP and NANPx6 target antigens that mAb 317 bound its target epitope with the greatest strength. Avidity differences between mAb 317 versus mAbs 580, CIS43, MGG4 and 311 were all statistically significant (Fig. 5; *p* values < 0.05). MAb CIS43 showed a disproportionately low avidity by ELISA against the NANPx6 peptide (Fig. 5B, D) which was consistent with its preference for the junctional region of CSP. Overall, a slower dissociation rate and a lower dissociation constant corresponded to higher avidity as was alluded to by Dennison et al*.*^33^ (*r* = − 0.71, *p* < 0.0001; Fig. S4C, D; Table S3).

### In vitro function

**Figure 6:**
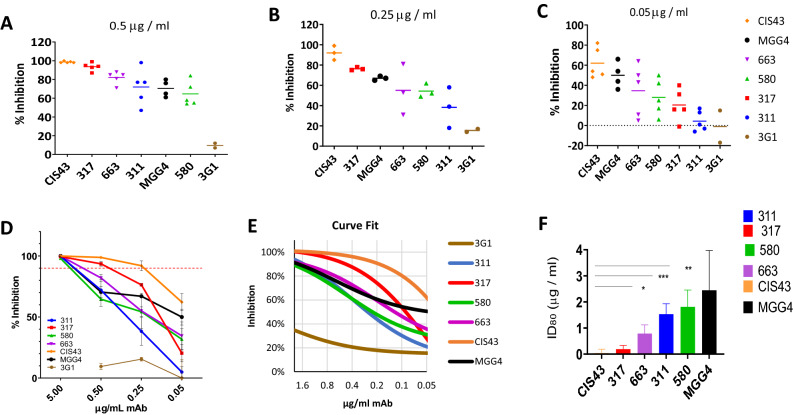
Inhibition of liver stage development assay (ILSDA). MAbs at varying dilutions were mixed with *P. falciparum* sporozoites (NF54 strain) and added to primary human hepatocytes. Hepatocyte invasion was quantified by RT-qPCR using a standard curve plotted using a pre-specified number of sporozoite mixed with the target cells. (**A**–**C**): Lines represent mean inhibition at (**A**) 0.5 µg/ml; (B) 0.25 µg/ml; (**C**) 0.05 µg/ml mAbs using data from three or more independent assays (symbols); negative control mAb 3G1 was run to show the effect of a non-specific mAb on sporozoite invasion. (**D**) Mean inhibition plotted against mAb concentrations (mean ± standard error) from 3 or more independent experiments; red line represents 80% inhibition. (**E**) Fitted inhibition curves used for the estimation of mAb dose required for 80% inhibition (ID_80_). (**F**) Estimated ID_80_ (mean + standard error) arranged in increasing order. Level of significance for pair-wise comparisons with mAb CIS43 are shown (*).

Inhibition of liver stage development assay (ILSDA) was performed using *P. falciparum* sporozoites and primary human hepatocytes. At 5 µg/mL, all CSP mAbs showed inhibitions approaching 100% (Fig. S5). Multiple inhibition assays at 0.5, 0.25 and 0.05 µg/mL were carried out to discern mAb-specific and dose-specific effects (Fig. 6A–C). Due to the intrinsic variability associated with batches of sporozoites and primary human hepatocytes, inhibition data from 3 or more independent ILSDA experiments for each mAb were fitted to a General Linear model and least square mean estimates were statistically compared (Table S4). Compared to the control mAb 3G1, at 0.5 and 0.25 µg/ml all the CSP mAbs inhibited invasion of *P. falciparum* sporozoites (Table S5; *p* values < 0.0001). CIS43, MGG4 inhibition was significantly higher than the control mAb even at the lowest dose level of 0.05 µg/ml (*p* < 0.0001) (Fig. 6C; Table S5). In pairwise comparisons between CSP mAbs, CIS43 displayed ~ 99% inhibition at 0.5 µg/ml that was significantly higher than mAbs MGG4, 311, 317, 580 and 663 (all *p* values < 0.05, Fig. 6A; Table S6). At 0.25 µg/ml, mAb CIS43 displayed 92% inhibition that was statistically superior to mAbs MGG4, 580, 663, 311 (Fig. 6B; Table S6, *p* values < 0.002). At 0.05 µg/ml dose mAb, CIS43 inhibition remained above mAbs 663, 580, 317 and 311 (Fig. 6C; Table S6; *p* values < 0.05). Overall, mAb 317 showed the second-best inhibition at 0.5 and 0.25 µg/ml concentrations, but at the lowest 0.05 µg/ml dose mAb MGG4 inhibited better than mAbs 317, 580 and 311 (Table S6; Fig. 6C, *p* values < 0.05).

The mAb dose response data (Fig. 6D) was fitted to a three-parameter Michaelis–Menten dose response model (Fig. 6E), allowing the estimation of mAb dose required for 80% inhibition (ID_80_) (Table S7; Fig. 6F). The ID_80_ value of the top performing mAb CIS43 (ID_80_ = 0.04 µg/ml; CI -0.26 to 0.33) was fivefold lower than mAb 317 (0.19 µg/ml; CI -0.1 to 0.48), although this ID_80_ difference was not statistically significant (Table S7). ID_80_ for mAb 580 showed it was 50 times less inhibitory than mAb CIS43 and 10 times less inhibitory than mAb 317 (*p* < 0.01).

### In vivo protection

Intravenous transfer of mAbs in C57BL/6 mice was followed by challenge with 1000 transgenic *P. berghei* sporozoites, expressing *P. falciparum* CSP, via the intravenous route^40^. The absence of blood stage parasites over the next 14 days indicated sterile protection. To discern differences between mAbs, contingency table analyses were performed by Fisher’s exact test and Kaplan-Meir survival curves were compared by a Log-rank test.

**Figure 7:**
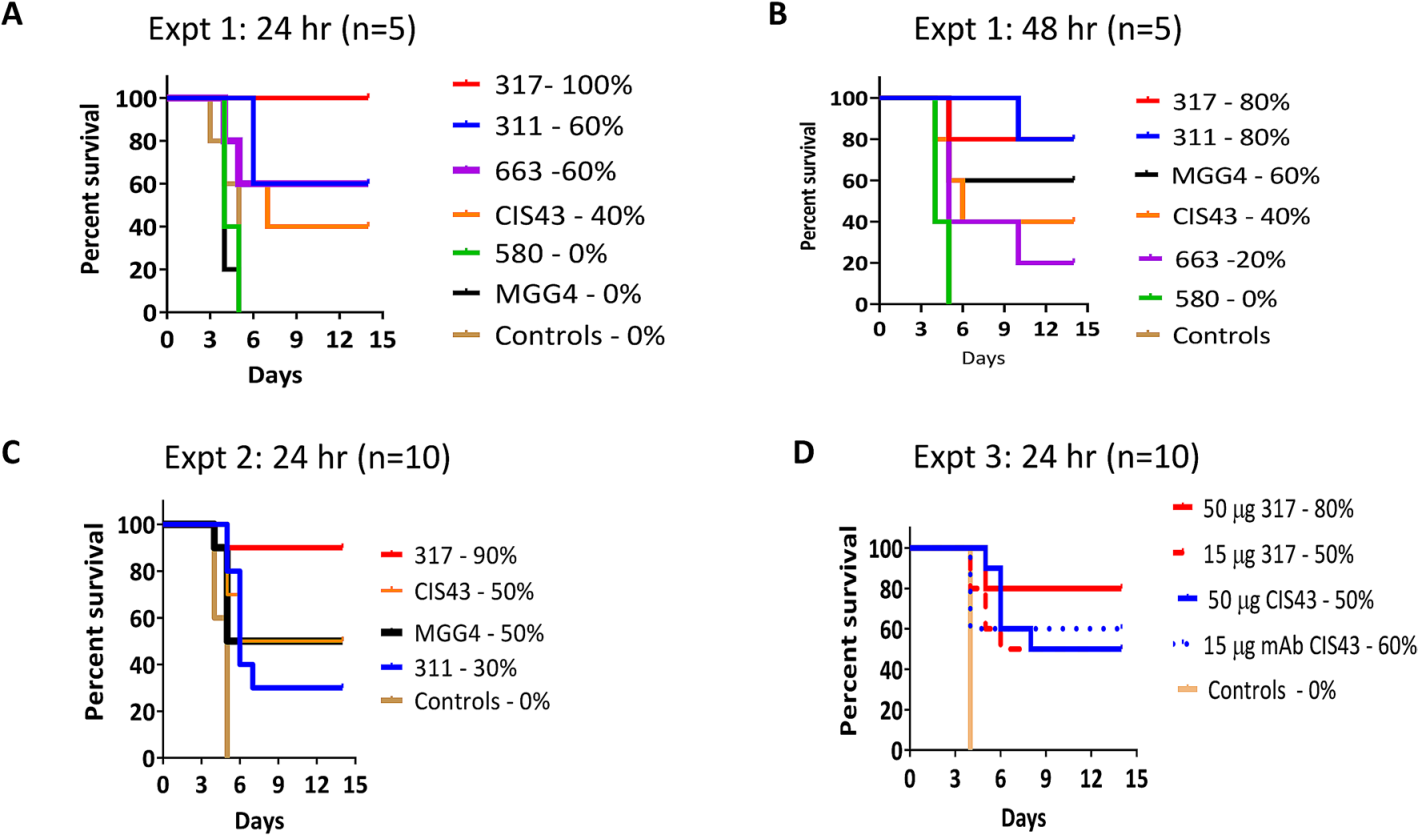
In vivo protection experiments. MAbs were passively transferred (i.v.) into mice followed by i.v. challenge with 1000 transgenic *P. berghei-falciparum* sporozoites. Survival curves and sterile protection outcomes at day-14 post challenge as determined by the absence of blood stage parasitaemia. (**A**) *Experiment 1* (100 µg each mAb; n = 5 per group) challenge at 24 h post transfer; (**B**) 48 h post challenge. (**C**) *Experiment 2* (100 µg mAb; n = 10) challenge at 24 h post mAb transfer. (**D**) *Experiment 3* using mAb 317 and CIS43 (50 and 15 µg dose; n = 10), challenge at 24 h post mAb transfer.

*Experiment 1:* 100 µg of each mAb was transferred and the mice were challenged at 24 h (n = 5) or 48 h (n = 5) post transfer (Fig. 7A, B). At 24 h, mAb 317 protected 100% mice compared to 0% of controls. MAbs 311 and 663 protected 60% mice and mAb CIS43 protected 40%. Fisher’s test showed higher frequency of protected mice for mAb 317 compared to the controls, mAb 580 and mAb MGG4 group (*p* values = 0.004) (Fig. 7A; Table S8). The five remaining mice were challenged at 48 h and mAb 317 showed 80% protection. MAb CIS43 protected 40% mice which was also consistent with the 24 h challenge. Unlike 0% and 30% protection for mAbs MGG4 and 311 in the 24 h challenge respectively, 60% and 80% protection were observed at 48 h challenge. The higher protection was likely due to the low mouse numbers (n = 5) and reduced sporozoite infectivity at 48 h challenge time-point. Fisher’s test on protection frequency for mAbs 311 and 317 groups showed significantly higher protection than the controls and mAb 580 (*p* values = 0.024). Log-rank test confirmed these inferences (Fig. 7B; Table S8).

*Experiment 2*: 100 µg mAbs 311, 317, CIS43, and MGG4 were transferred (n = 10 per group) and mice were challenged at 24 h post transfer (Fig. 7C; Table S8). Compared to no protection in the controls, mAb 317 showed the best protection (90%) and CIS43 protected half of the challenged mice (50%). MAb MGG4-mediated protection (50%) was consistent with the 48 h challenge result from *Experiment 1*. MAb 311 protection (30%) was lower than mAb 317 similar to that observed in the 24 h challenge of *Experiment 1*. Protection frequency compared by Fisher’s test showed mAb 317, CIS43 and MGG4 protection were higher than the controls (all *p* < 0.02), and mAb 317 protected significantly better than mAb 311 (*p* = 0.01). Log-rank test had consistent inferences (Table S8).

*Experiment 3*: MAbs 317 and CIS43 were tested head to head at 50 and 15 µg (n = 10 per group) and mice were challenged at 24 h post transfer (Fig. 7D). Compared to no protection in the controls, mAb 317 showed 80% and 50% protection while mAb CIS43 protected 50% and 60% mice in the high and low dose groups respectively. Fisher’s test showed both mAbs displayed significantly higher protection than the controls. At this lower dose, no difference in protection outcome between mAbs or dose levels was observed (Table S8). Log-rank test had consistent inferences.

Overall, the control mice were always infected, mAb 317 showed the best protection and mAb CIS43 protected about half the mice even at the lower doses.

### Sporozoite binding assay

**Figure 8:**
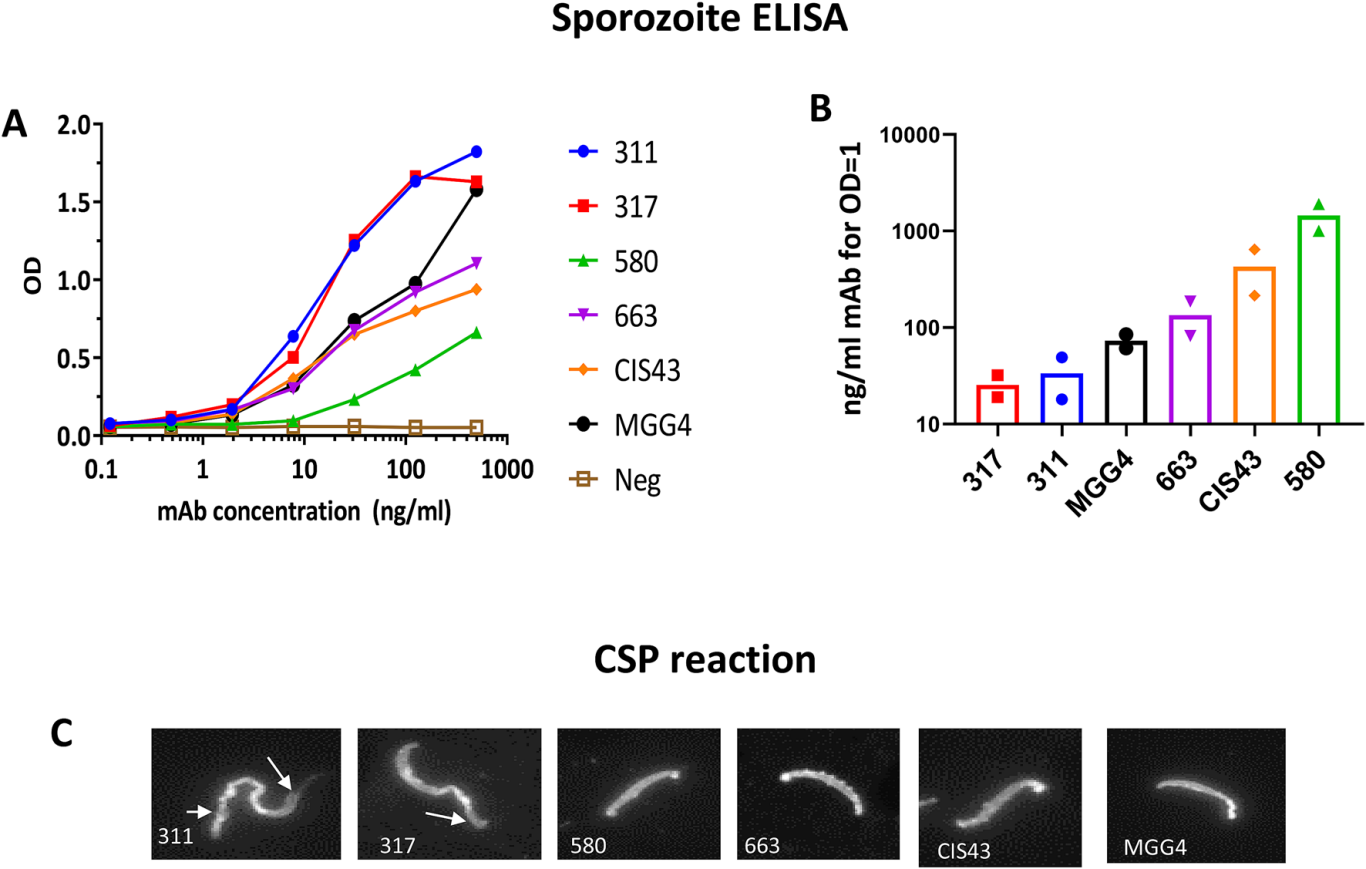
MAb reactivity to sporozoite. (**A**) ELISA curves of mAbs binding to transgenic *P. berghei* sporozoites carrying the *P. falciparum* CSP gene. (**B**) Mean ng/ml that resulted in OD = 1 for the sporozoite ELISA. Data from two independent experiments are shown as symbols. (**C**) Representative CSP reaction assay at 100 ng/ml mAb, performed twice using live transgenic *P. berghei* sporozoites carrying a functional *P. falciparum* CSP gene. Arrows indicate a typical tail-like CSP reaction at the ends of sporozoites.

To study the interaction of the mAbs with the transgenic sporozoites used during the challenge studies, a direct ELISA was performed against transgenic sporozoites. In two independent experiments, mAbs 317 and 311 bound to the sporozoites more efficiently than mAbs MGG4, 663 and CIS43, while mAb 580 bound least efficiently (Fig. 8A, B). CSP mAb binding to live sporozoites can cause a CSP reaction observed microscopically as a tail-like precipitate^41,42^. MAbs 311 and 317 (at 100 ng/mL) showed a positive CSP reaction, whereas no clear reaction was observed at this concentration with the other CSP mAbs (Fig. 8C).

### Associations with in vivo protection

An exploratory Pearson’s correlation analysis was performed on raw in vitro data and the corresponding in vivo protection frequency from independent challenges performed as part of *Experiment-1* and *2* (Table S3). Several strong (correlation coefficients |*r*|> 0.7) to moderate linear associations (|*r*|= 0.4–0.7) are listed in Table 2. Direct ELISA reactivity (ng/ml mAb needed for OD = 1) against a short repeat peptide (NANPx6), showed a consistent negative association. Inhibition of sporozoite invasion, measured by ILSDA at 0.5 µg/ml, showed a consistent positive association. Strong and consistent associations were found with FL-CSP and NANPx6 avidity assays; likewise, strong negative associations were observed for *K*_D_ values, measured by BLI using bivalent and Fab fragments*.* Finally, an estimate of the size of the interface between mAbs and their epitopes called the Buried Surface Area (BSA) has been shown to be associated with CSP mAb affinity^19^. We calculated BSA from mAb crystal structures (Fig. 1) using AREAIMOL program^43^ and found a strong positive association with protection. The most protective mAb 317 showed the highest BSA (> 500 Å^2^).

**Table 2:** Correlation analysis.

Protection study	Expt 1 (24 h)	Expt 1 (48 h)	Expt 2 (24 h)
ELISA (FL-CSP)	0.27 (0.30)	**0.75 (0.00)**	0.26 (0.28)
ELISA (NANPx6)	− 0.54 (0.14)	− **0.73 (0.01)**	− 0.14 (0.58)
ELISA (SPZ)	− 0.47 (0.21)	− 0.60 (0.08)	0.46 (0.02)
ILSDA (0.5 µg/ml)	0.54 (0.00)	0.41 (0.11)	**0.71 (0.01)**
Avidity Index FL-CSP	**0.72 (0.01)**	0.68 (0.01)	0.59 (0.03)
Avidity Index NANPx6	0.68 (0.02)	**0.78 (0.00)**	0.67 (0.02)
Avidity (FL-CSP)	**0.77 (0.00**)	0.53 (0.12)	**0.84 (0.00)**
Avidity (NANPx6)	0.67 (0.03)	**0.77 (0.00)**	0.65 (0.02)
*K*_D_ Bivalent (FL-CSP)	− 0.55 (0.18)	− **0.80 (0.01)**	− 0.26 (0.46)
*K*_D_ Fab (FL-CSP)	− 0.56 (0.25)	− **0.82 (0.05)**	− 0.14 (0.86)
*K*_D_ Fab (NANP6)	− 0.56 (0.25)	− **0.73 (0.10)**	− 0.34 (0.66)
BSA (NPNA)_n_	**0.84 (0.04)**	**0.81 (0.05)**	0.36 (0.64)
BSA junctional	0.65 (0.17)	0.59 (0.22)	0.20 (0.80)

Pearson’s correlation coefficient *r* and the level of significance (bracket) between in vivo challenge (*Experiment 1* and *2*) and in vitro assays. Assays included FL-CSP ELISA; NANPx6 ELISA; Sporozoite ELISA; ILSDA at 0.5 µg/ml; Avidity Index (AI) against FL-CSP; AI against NANPx6; Avidity against FL-CSP; Avidity against NANPx6; *K*_D_ Bivalent against FL-CSP; KD Fab against FL-CSP; *K*_D_ Fab against NANP6; Buried Surface Area between mAb and (NPNA)n epitope and BSA against junctional epitope. Pearson's correlation coefficient |*r*|> 0.7 suggested a strong linear association; |*r*|= 0.4–0.7 suggested moderate linear association; and no linear association was inferred for |*r*|< 0.4. Bold values are |*r*|> 0.7 (*p* < 0.05).

## Discussion

Short unstructured peptides are not particularly immunogenic due to a conformational entropy cost of stabilizing flexible epitopes^44,45^, but the repetitive and flexible repeat region of CSP is quite immunogenic^16^. In addition to cross-linking the B-cell receptors^15^, NPNA, DPNA and NPNV repeats are rich in asparagine residues whose side-chain has lower flexibility and a propensity to hydrogen bond back to the main chain^46^. This narrows the main chain flexibility of CSP repeats to the terminal alanine and valine residues between the short structured motifs: like the type I and ‘3_10_′ turns^32^. As a result, the partly constrained (NPNA)_n_ and the junctional sequence can bind an assortment of antibodies by an induced fit process^47–49^. Six such repeat mAbs were tested head-to-head, in immuno-chemical, biological and challenge experiments with transgenic *P. berghei* expressing *P. falciparum* CSP. Since this study was initiated, other potent *P. falciparum* CSP mAbs have been reported from human recipients of PfCVac, PfSPZ vaccine and recombinant CSP vaccinated transgenic mice (Kymab)^32,50,51^. While some recently reported mAbs were not included, the comparative biophysical and immunological assessment of 6 mAbs with a wide range of affinities provided key insights into CSP antibody effectiveness and function.

*First,* we found that steady-state assays with long NPNA repeats (like CSP ELISA) were not predictors of protective efficacy likely due to the flexibility of the target repeat epitope. *Second,* mAb 317 whose Fabs and bivalent forms showed the highest affinity were most protective in vivo; while mAb 580 with the lowest affinity, originally derived from an IgM^19^, was not protective. The high affinity of mAb 317 was driven primarily by its low dissociation rate. *Third*, antibodies like CIS43 bound to the full-length CSP with higher affinity than the NANPx6 peptide, suggesting targets outside the major (NPNA)_n_ repeats were also important. *Forth,* bivalent forms of some lower-affinity mAbs (such as 663, MGG4, 580) bound more strongly than their respective Fab fragments. While Fab affinity plays an important role in repeat antibody mediated protection, cooperative interactions between Fabs do not always augment immune-protection^20,24^.

The complex interplay between Fabs and intact IgG binding to CSP reported here has important implications for vaccine design. For example, a blood stage merozoite enters a RBC within a minute^52^, but a sporozoite transit from skin to the liver can take up to 30 minutes^53^. We hypothesize that CSP retains the highly repetitive and flexible central region during evolutionary selection to allow certain low affinity antibodies to bind and compete with the truly high affinity antibodies, diluting the overall effectiveness of host immunity. Mechanistically a bivalent antibody, but not Fab fragments, precipitate CSP on sporozoites^54^. Lower affinity bivalent mAbs have a lower tendency to precipitate CSP as they may be coalescing along a single CSP molecule through homotypic interactions. In contrast, higher affinity mAbs may be cross-linking adjacent CSP molecules disrupting motility and impairing sporozoite cell traversal. In future experiments it may be interesting to engineer vaccines that elicit antibodies (Fabs) that use multiple weak interactions to bind to CSP and compare them to vaccines that promote 317-like antibody properties. Indeed, restricting epitope flexibility using short loops displayed on the Tobacco Mosaic Virus-like particle has been shown to improve immunogenicity, avidity and protective efficacy of a CSP repeat-based vaccine^16^.

Compared to the standard dose regimen, RTS,S delayed and fractional third dose (DFD) resulted in higher somatic hypermutation, avidity, and protection in humans^8^. Not surprisingly, the RTS,S-induced mAb 317 displayed the highest affinity within the panel, it showed efficient sporozoite binding by ELISA, a positive CSP reaction and low ID_80_ < 0.5 µg/mL. Most importantly, mAb 317 protected 100% of mice at ~ 5 mg/kg (100 µg in a 20 g mouse) which is within the therapeutic dose range recommended for prophylactic mAb products against Ebola (Zmapp; ~ 50 mg/kg^55^) and Respiratory Syncytial Virus (Palivizumab; ~ 15 mg/kg^56^). In our model, mAb 317 protection dropped to 80% and 50% at 50 µg and 15 µg doses. Others have also reported mAb 317 protection to wane from 100% (at 300 μg) down to 80% and 0% (at 100 or 30 μg respectively)^32,57^. Like RTS,S, the efficacy of a NANP-specific prophylactic mAb may wane rapidly as the circulating mAb concentration drops. Overall, the RTS,S mAb 317 emerged as prototype of a highly protective mAb in our mouse protection model; mutations to improve its half-life and serum retention need to be evaluated.

Previously mAb CIS43 was shown to protect 100% of mice at 300 µg dose against transgenic parasite challenge along with potent inhibition of liver stage development in vitro^12^. In our studies, mAb CIS43 consistently protected about half of the challenged mice at 100 µg dose, despite being the most potent mAb in the ILSDA assay. The lower in vivo protection of mAb CIS43 (compared to mAb 317) was reflected in its 20-fold reduced Fab affinity and low avidity for the NANPx6 peptide. A biological explanation for the disconnect between the in vitro and in vivo readouts for mAb CIS43 could be that the transgenic parasites do not fully recapitulate the molecular events that occur between *P. falciparum* sporozoites and human hepatocyte^6^. Alternatively, a fraction of transgenic sporozoites may be resistant to CIS43 mediated inhibition in vivo. Tan et al*.* and Kisalu et al*.* have hypothesized that junctional sequence mAbs have biological activities in addition to inhibition of sporozoite motility and traversal^12,21^. We also observed that a junction epitope cross-binder mAb MGG4 had the second-highest ILSDA activity (after mAb CIS43) at the lowest mAb concentration tested (0.05 μg/mL). A clinical trial for mAb CIS43 containing a half-life extension mutation^58^ is reported to be underway using 5, 20, and 40 mg/kg doses and our ILSDA and efficacy data supports determining the protective role of junctional vaccines and mAbs in humans^51^.

Despite the impressive biological activity reported for some cross-reactive mAbs^12^, Oyen et al*.* showed that a cross-reactive mAb 668 was less protective than the (NPNA)_n_-specific mAb 317^32^. A definitive study using 200 mAbs (that included mAb CIS43 and 317), Murugan et al*.* concluded that high affinity, and not epitope specificity was the primary driver of repeat mAb protection and in vitro function^22^. In agreement with Murugan et al*.*, we found that protection outcome was highly dependent on mAb affinity and avidity readouts. While we cannot assign a cut-off affinity for a highly protective antibody, mAb 317 emerged as an excellent benchmark for future attempts to improve anti-CSP affinity. A positive correlation between BSA and protection observed here suggested that in silico directed modifications may be useful in improving CSP mAbs. Extremely high affinity can however present other liabilities. For example, the RSV prophylactic Motavizumab, that had higher affinity and neutralization profile than Palivizumab (Synagis; Medimmune), also showed an unforeseeable non-specific binding to human tissue^59^. Indeed, a report on differences in circulating serum concentrations of some CSP mAbs caution against off-target binding of CSP mAbs (e.g. the cross-binder mAb 4493)^22^.

The WRAIR intravenous challenge model bypasses skin immunity^60^ and protection may rely on a multitude of anti-parasitic effects of CSP antibodies including inhibition of motility and traversal^61^, Fc mediated opsonization^62^, complement mediated killing^63^, blocking of proteolytic processing of CSP^64^, or blocking of a putative receptor-ligand interaction^65^. This model measures protection in the blood which requires inactivation of 100% of the parasites in the inoculum and is sensitive to the biological and technical variability between challenge experiments for example the variation in protection outcomes between 24 and 48 h challenge with mAbs MGG4 and 311. While we used only one of several transgenic parasite lines and in vivo models available^66,67^, our data showed mAb 317 consistently elicited the highest protection at 100 µg dose across 3 independent challenges (100%, 80%, 90%) and mAb CIS43 consistently protected about half the mice (40%, 40%, 50%). Our data provide support for a CHMI trial comparing efficacy of a high affinity (NPNA)_n_-specific mAb (like 317) and a potent dual-specific mAb (such as CIS43). Furthermore, the associations between in vivo protection and immune-chemical assays reported here call for prioritizing affinity and avidity assays on Fab and bivalent mAbs in addition to ILSDA and in vivo challenge for the down-selection of future CSP-based interventions.

## Methods

### Production of monoclonal antibodies

Antibody fragment (Fab) sequences for monoclonal antibodies (mAbs) 311, 317, CIS43, MGG4, 580, and 663 were obtained from the literature and synthesized with a human IgG Fc sequence^12,18,19,21^. Plasmids containing codon optimized full-length IgG genes (ATUM, Newark, CA) were transformed into Top10 *E. coli* cells (Thermo Fisher, Waltham, MA). Plasmid DNA was harvested and purified using the EndoFree Plasmid Purification Kit (Qiagen, Germany) following the manufacturer’s instructions. Equal amounts of heavy chain and light chain DNA were transfected into HEK293 cells, followed by antibody purification using Protein G (GE healthcare). Protein purity was confirmed by SDS-PAGE under reduced and non-reduced conditions (Fig. S1).

### Western blot

*Plasmodium falciparum* sporozoites (NF54 strain ~ 20,000 per well) were separated using SDS-PAGE gel and transferred onto nitrocellulose membranes using the IBlot system (Thermo Fisher, Waltham, MA). Membranes were blocked with casein overnight at 4 °C and incubated with diluted mAb (1 µg/mL) for 1 h. Blots were washed three times with PBS-Tween solution (1X PBS + 0.05% Tween) and Goat anti-human antibody conjugated to Alkaline phosphatase (1:5000) was added and incubated for 1 h. Following the final wash NBT/BCIP substrate was added to visualize the bands.

### Immunofluorescence assay

Slides were prepared by adding 10,000 *P. falciparum* sporozoites diluted in 1% BSA/RPMI per well and fixed with methanol. After blocking with 1% goat serum for 30 min, mAbs were added and allowed to incubate in a closed container for 1 h at room temperature. Samples were aspirated off and wells washed thrice with PBS solution. FITC-conjugated secondary antibody (1:500) was added at room temperature for 1 h and cover slip was mounted in mounting media. Fluorescence and phase images were photographed under 40 × magnification.

### ELISA

Immulon 4HBx 96-well plates (Thermo Fisher, Waltham, MA) were coated overnight with 100 ng/well of the following peptides at 4 °C: full-length FL-CSP, NANPx6, peptide 21 (NPDPNANPNVDPNAN)^12^, or NPDP_19_ peptide (KQPADGNPDPNANPNVDPN)^21^. Plates were washed thrice with PBS-Tween (1X PBS + 0.05% tween) solution and blocked for 1.5 h at room temperature with 0.5% casein + 1% Tween solution. MAbs were plated starting between 1000 and 200 ng/ml and serially diluted threefold down the plate. After 2 h incubation at room temperature, unbound antibodies were washed away thrice with PBS-Tween solution and remaining mAbs were detected using Anti-human IgG-horseradish peroxidase (HRP) conjugate (1:5000; Southern Biotech, Birmingham, AL) as secondary antibody along with 100 µl per well of ABTS 2-component substrate (KPL, Gaithersburg, MD) for color development for 1 h. After stopping the reaction with 20% SDS, optical density at 415 nm (OD_415_) was measured on a Synergy 4 microplate reader (BioTek, Winooski, VT). Concentration of mAb needed to achieve an OD of 1 using Gen5 software (BioTek, Winooski, VT).

### Whole sporozoite ELISA

The assay was essentially performed as described previously^68^. Transgenic *P. berghei* sporozoites expressing *P. falciparum* CSP were added to Immulon 2HB 96-well plates (Thermo Fisher, Waltham, MA) at a concentration of 25,000/well and incubated at room temperature for 2 h. Plates were fixed with methanol and blocked with 1% BSA/PBS for 1 h. MAbs were serially diluted fourfold down plates starting at 2 µg/mL and incubated at room temperature for 2 h. After washing thrice with PBS, the assay proceeded similarly as described above.

### Avidity index ELISA

A set of two (in duplicate) Immulon 4HB 96-well plates (Thermo Fisher, Waltham, MA) were coated with 100 ng/well of full-length FL-CSP or NANPx6 peptide at 4 °C. Plates were washed thrice with PBS-Tween solution and blocked for 1.5 h at room temperature with 0.5% casein + 1% Tween solution. MAbs were plated between 1000 and 200 ng/ml and serially diluted threefold down the plate. After 2 h incubation, unbound antibodies were washed and 50 µl/well of 2 M Sodium thiocyanate (SCN) was added to one of the two plates, and the other plate received 50 µl/well PBS for 15 min. After washing with PBS-Tween solution, the assay proceeded similarly as described above. MAb titer was calculated as the concentration of mAb needed to achieve OD = 1 using a 4-parameter curve fit on Gen5 Software (BioTek, Winooski, VT). Avidity index (%) was determined as mAb titer (ng/ml) on the PBS plate / thiocyanate plate.

### Thiocyanate dilution ELISA

Immulon 4HB 96-well plates (Thermo Fisher, Waltham, MA) were coated with 100 ng/well of FL-CSP protein or NANPx6 as described above. Plates were washed and blocked with 200 µl of 0.5% Casein-PBS for 2 h at room temperature. Plates were washed with PBS-Tween solution and mAbs at 100 µl of 200 ng/ml were plated 2 h at room temperature. Unbound antibodies were washed starting from the top well with 100 µl of aqueous SCN solutions (0 M, 1 M, 2 M, 3 M, 4 M, 5 M, or 6 M) for 15 min at room temperature. After washing with PBS-Tween, the assay proceeded similarly as described above. Linear regression was used to determine the concentration of NaSCN that reduced OD_415nm_ to half maximal of the OD in the 0 M NaSCN wells.

### Fab binding kinetics

Fab fragments were produced by incubating human mAbs (1 mg/mL) with Endoproteinase Lys-C at a ratio of 1:2500 antibody:enzyme at 37 °C for 2–3 h. Enzyme digestion was assessed by SDS-PAGE and upon completion, the reaction mixture was passed through Protein-A beads (0.5–1 mL beads) three times. The final flow through was collected and assessed by SDS-PAGE for purity. Affinity measurements were performed using an Octet RED96 (ForteBio, Freemont, CA) instrument. *P. falciparum* FL-CSP protein was biotinylated using the EZ-link sulfo-NHS-biotin reagent (Thermo Scientific, Waltham, MA) following manufacturer’s instructions, while biotinylated NANPx6 was obtained commercially (Atlantic peptides, Lewisburg, PA). Kinetics buffer (ForteBio, Freemont, CA) was used for all dilution, baseline, and dissociation steps. Streptavidin biosensors, were loaded with CSP protein (50 µg/mL; response level: 1.2–1.5 nm) or NANPx6 peptide (30 µg/mL; response level: 4 nm) and dipped into wells containing dilutions of prepared Fabs (1.06–0.013 nM) for 20–90 s and were then allowed to dissociate for 10–350 s in buffer ensuring at least 5% dissociation. After reference subtraction, binding kinetic constants were determined using at least four concentrations of Fab by fitting the curves to a 1:1 Langmuir binding model (Table S2) using data analysis software 9.0 (ForteBio, Freemont, CA).

### Bivalent antibody kinetics

Affinity measurements of bivalent mAbs were determined by biolayer interferometry as previously described using Octet RED384 instruments and biosensors (ForteBio, Freemont, CA)^33^. Briefly, the full length FL-CSP was coupled to amine reactive (AR2G) biosensors with threshold set not to exceed 0.1 nm. Ovalbumin coupled (at similar density) AR2G sensors were used as parallel reference sensors. To monitor antibody association, antigen-loaded sensors were first dipped into wells containing 1 × kinetics buffer (ForteBio) for baseline measurement, then dipped into wells containing mAb in 1 × kinetics buffer at various concentrations. Antibody-bound sensors were dipped back into wells used for baseline measures to monitor dissociation. Kinetic assays were all performed at 25 °C using standard settings. The specific binding courses of 4 to 7 concentrations of antibodies were fitted to a 1:1 Langmuir binding model using Data Analysis Software 9.0 (CFR11) to obtain the rate constants and KD values. The mean values of quadruplicate binding measurements made using two instruments are reported (Table S2).

### Inhibition of liver stage development assay (ILSDA)

The ILSDA was performed as previously described^30^. Briefly, *P. falciparum* (NF54 strain) sporozoites were incubated with NFS1 (positive control mAb) or human mAb at room temperature for 20 min. A primary human hepatocyte culture (BioReclamation IVT, Baltimore, MD) was inoculated with the sporozoite-antibody mixture and incubated at 37 °C for 3 h to allow sporozoites to infect hepatocytes. The culture was washed following the first incubation period to remove non-invaded sporozoites, and washed again at 24 h post-inoculation. Total RNA was isolated from hepatocytes 72 h after the second wash and purified for downstream quantitative real-time PCR (qRT-PCR) of *Pf* 18S rRNA. RNA levels were quantified in quadruplicate to determine the percent inhibition of liver stage development by each antibody compared to negative control (no antibody). To generate the standard curve, fixed number of Pf sporozoites (20. 60, 180, 540, 1620, 4860 were added to the wells seeded with cryopreserved primary human hepatocytes, the mixture was harvested and RNA are extracted for downstream RT-PCR analysis. Unknown sample parasite burden was determined using a standard curve between CT values and sporozoite. A representative standard curve used for a mAb ILSDA experiment is shown in Fig. S6. ILSDA experiments used to compare mAbs (0.5, 0.25 and 0.05 µg/ml) were repeated at least 3 times.

### In vivo protection

All procedures were reviewed and approved by the Walter Reed Army Institute of Research’s Animal Care and Use Committee and performed in accordance with the Animal Welfare Act and other federal statutes relating to animals and experiments. The study was carried out in compliance with the ARRIVE guidelines (http://www.nc3rs.org.uk/page.asp?id=1357). Female C57BL/6 J mice (The Jackson Laboratory, Bar Harbor, ME) were intravenously injected with 100 µL of each mAb in sterile PBS. Sterile protection of animals by mAb was assessed using transgenic *P. berghei* sporozoites expressing functional, full-length *P. falciparum* CSP^40,66^. Animals were challenged 24 or 48 h post mAb transfer with 1000 sporozoites intravenously. Blood-stage parasitaemia was detected by microscopy of giemsa-stained thin blood smears. Percent survival was determined using the absence parasitaemia during the two-week observation period immediately following challenge. Fisher’s exact and Log-rank tests were performed to compare survival outcomes and curves for each experiment, respectively.

### CSP reaction

Biotinylated mAbs at 1000, 100 and 10 ng/mL were mixed with freshly dissected transgenic *P. berghei* sporozoites for 10 min at room temperature. The antibody-sporozoite mixtures were placed drop-wise onto microscope slides, fixed with methanol, and allowed to air dry. Slides were blocked with casein and incubated with an Avidin-FITC secondary antibody (Thermo Fisher, Waltham, MA) before being visualized at 40X under a Fluorescence microscope (Olympus). A positive sporozoite reaction was recorded if tail-like projections were seen on mAb incubated sporozoites. A negative control mAb 3G1 was used to observe for background reaction.

### Statistical methods

All the raw data used in statistical analyses is shown in Table S9. Repeat measurements on sporozoite invasion inhibition by ILSDA were modeled using a Generalized Linear Model (GLM). Generalized Estimating Equation (GEE) was used to determine the effect of dose and mAb type on inhibition of sporozoite invasion. Least Square (LS) means (Table S4); pairwise comparison of LS means against negative control mAb 3G1 (Table S5), and paired comparison between the CSP mAbs (Table S6) were used to discern statistically significant effects. The ILSDA inhibition data were fitted to a three-parameter Michaelis*–*Menten model $\left( {{\text{ f}}\left( {\text{x}} \right) = {\text{c}} + \frac{{{\text{d}} - {\text{c}}}}{{1 + \frac{{\text{e}}}{{\text{x}}}}} + {\upvarepsilon }} \right)$^69^ and F test was used to determine if regression parameters were equal and whether fitted curves coincide. Fitted curves from the equation were shown in Fig. 6E. The estimated inhibitory mAb dose needed for 80% inhibition from the fitted model were listed in Table S7. In vivo protection data on day 14 were analyzed by contingency table analyses. Fisher’s Exact test (Table S8) was used to examine the difference in infection rates. Kaplan Meir survival curves were analyzed using both the log-rank and Wilcoxon tests and showed similar *p* values. Paired association analyses between in vitro assays and in vivo protection was performed using raw data on 18 variables. Linear regression or Generalized Linear model, was applied with and without repeated observations. The standardized regression coefficient was equivalent to the Pearson’s Correlation coefficient for each pair-wise comparison and *p* values (Table S3). For all other comparisons, one-way ANOVA was used and *p* values corrected for multiple comparisons using the Tukey’s method. *P*-values less than 0.05 were considered to be significant. We analyzed the data using SAS/STAT Software, Version 9.4 of the SAS System TS1M5 (Cary, NC). Graph Pad Prism 8 (GraphPad Prism software, La Jolla, CA) and Microsoft Excel were used for plotting.

### Ethics approval

Animal procedures were conducted in compliance with the Animal Welfare Act and other federal statutes and regulations relating to animals and experiments involving animals and adhere to principles stated in the Guide for the Care and Use of Laboratory Animals, NRC Publication, 2011 edition.

## Supplementary information

Supplementary information.

## Data Availability

The datasets and reagents used during the current study are available from the corresponding author on reasonable request.
