# Supplementary material for: In vitro and in vivo inhibition of malaria parasite infection by monoclonal antibodies against Plasmodium falciparum circumsporozoite protein (CSP)
Source: Sci Rep. 2021 Mar 5;11:5318. doi: 10.1038/s41598-021-84622-x (PMC7970865; doi:10.1038/s41598-021-84622-x)
Supplement: Supplementary file 1 — Supplementary information. [file 41598_2021_84622_MOESM1_ESM.docx]

***In vitro* and *in vivo* inhibition of malaria parasite infection by monoclonal antibodies against *Plasmodium falciparum* Circumsporozoite protein (CSP)**

Merricka C. Livingstone^1^, Alexis A. Bitzer^1^, Alish Giri^1^, Kun Luo^1^, Rajeshwer S. Sankhala^3,4^, Misook Choe^3,4^, Xiaoyan Zou^5^, S. Moses Dennison^6,7^, Yuanzhang Li^7^, William Washington^7^,Viseth Ngauy^2^, Georgia D.Tomaras^6,7,8,9,10^, M. Gordon Joyce^3,4^, Adrian H. Batchelor^1^ and Sheetij Dutta^1^.

^1^Structural Vaccinology Lab, ^2^Malaria Biologics Branch, Walter Reed Army Institute of Research, Silver Spring, Maryland, USA.

^3^US Military HIV Research Program, Walter Reed Army Institute of Research, Silver Spring, Maryland, USA.

^4^The Henry M. Jackson Foundation for the Advancement of Military Medicine, Bethesda, Maryland, USA.

^5^Malaria Department, Naval Medical Research Center, Silver Spring, MD, 20910;

^6^Center for Human Systems Immunology, Departments of ^7^Surgery, ^8^Immunology, ^9^Molecular Genetics and Microbiology, ^10^Duke Human Vaccine Institute, Duke University Medical Center, Durham, North Carolina, United States of America.

^7^Statistics and Epidemiology Branch, Walter Reed Army Institute of Research, Silver Spring, Maryland, USA.

**Supplementary Fig S1:** Monoclonal antibody (mAb) purity. SDS-PAGE gel showing non-reduced samples (left) and reduced samples (right).

**
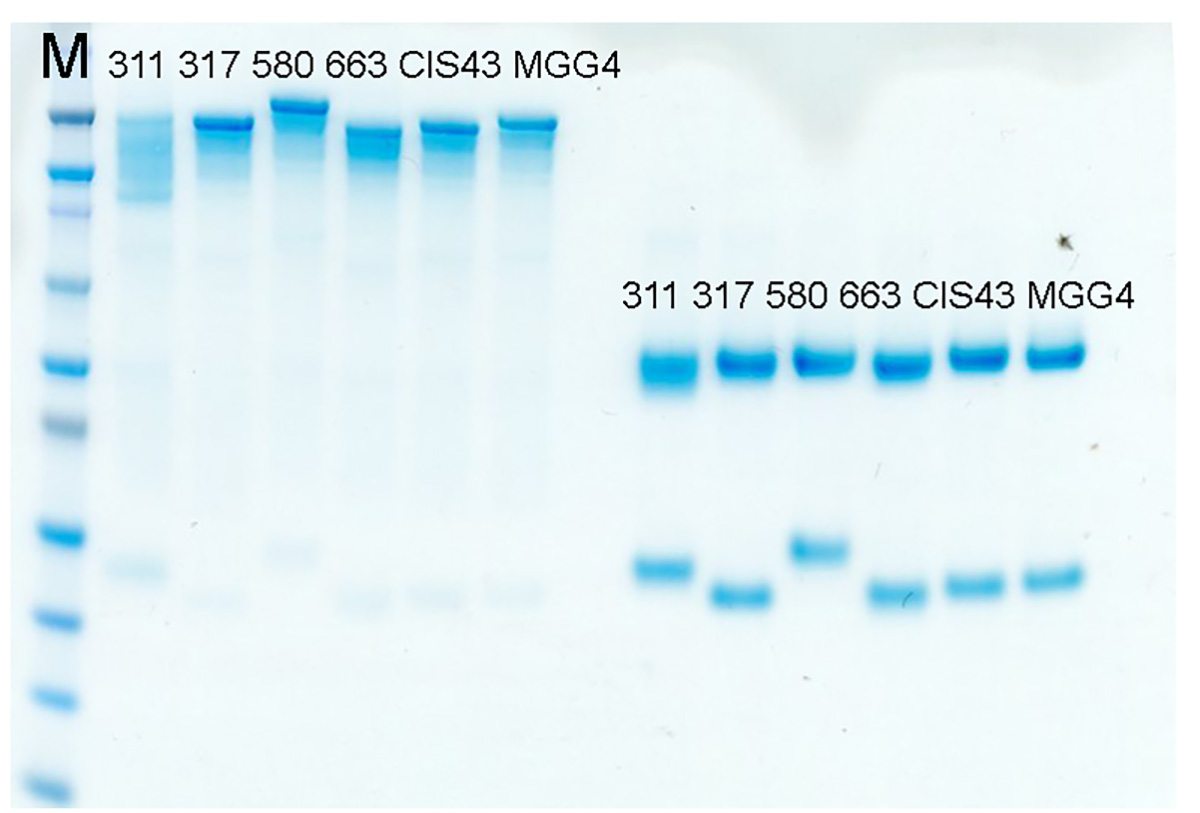
**

**Supplementary Fig. S2:** Global fit to a 1:1 Langmuir binding model for Fab binding to the NANPx6 peptide, performed twice on at least four concentrations of Fabs. Analysis was conducted on blinded samples at the Structural Biology Lab, Military HIV Research Program, WRAIR.

**Supplementary Figure S3:** Global fit to a 1:1 Langmuir binding model for Fab binding to the FL-CSP; performed twice on at least four concentrations of Fabs. Analysis was conducted on blinded samples at the Structural Biology Lab, Military HIV Research Program, WRAIR.

**Supplementary Figure S4:** Avidity and affinity correlations. Positive association between avidity determined by 2M NaSCN wash method (*y-axis*) or determined using serial NaCSN dilution wash method (*x-axis*) against (A) FL-CSP target antigen or (B) NANPx6 target peptide. (C) Negative association between dissociation rate constant (*k*_d_) and avidity index against FL-CSP target antigen. (D) Negative association between equilibrium dissociation constant (KD) and avidity index against FL-CSP target antigen.

**Supplementary Figure S5.** ILSDA. Mean inhibition of sporozoite invasion at 5 µg/ml mAb in two or more independent experiments (symbols).

**Supplementary Figure S6:**  A representative RT PCR standard curve generated during one of the ILSDA assays showing a linear relationship between CT values (*y-axis*) and pre-specified number of sporozoites (red symbols on the *x-axis*).

**
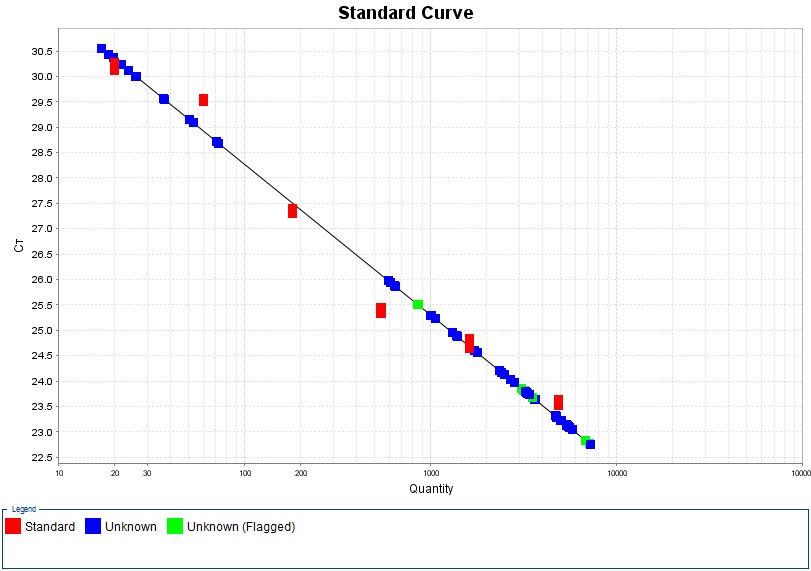
ite**

**Table S1.** Summary of published mAb affinity data with method used and references. ITC, isothermal titration calorimetry; BLI, biolayer interferometry; SPR, surface plasmon resonance.

| **Fab** | **Antigen** | **Affinity** | **Method** | **Ref** |
| --- | --- | --- | --- | --- |
| 311 | (NPNA)_2_ | 296 nM | ITC | ^18^ |
|  | (NPNA)_3_ | 305 nM |  |  |
|  | (NPNA)_2_ | 160 nM |  |  |
|  | NPNVDPNANPNV | 1.79 µM |  |  |
|  | DPNANPNVDPNA | 1.37 µM |  |  |
| 317 | (NPNA)_2_ | 173 nM | ITC | ^18^ |
|  | (NPNA)_3_ | 78 nM |  |  |
|  | (NPNA)_2_ | 135 nM |  |  |
|  | NPNVDPNANPNV | 12.1 µM |  |  |
|  | DPNANPNVDPNA | 448 nM |  |  |
|  | KQPADGNPDPNANPNV | no binding | BLI | ^32^ |
|  | (NPNA)_4_ | 25 nM |  |  |
|  | NP(NANP)_5_ | <0.1 nM | SPR | ^22^ |
|  | NVDPNANPNVDPNANPNVDP | 0.1-1 µM |  |  |
|  | NPDPNANPNVDPNANP | ~1 µM |  |  |
| CIS43 | pep 21 (NPDPNANPNVDPNAN) | 7.5 nM | ITC | ^12^ |
|  | pep 29 (NANPNANPNANPNAN) | 53 nM |  |  |
|  | rPfCSP | 0.91 nM | BLI |  |
|  | pep 21 (NPDPNANPNVDPNAN) | <0.001 nM |  |  |
|  | pep 29 (NANPNANPNANPNAN) | 4.66 nM |  |  |
|  | NP(NANP)_5_ | 0.1-1 µM | SPR | ^22^ |
|  | NPDPNANPNVDPNANP | 1-10 nM |  |  |
|  | NVDPNANPNVDPNANPNVDP | 10 nM |  |  |
| 580 | CSP | 1.09 µM | BLI | ^19^ |
|  | (NANP)_5_ | 1.47 µM | SPR |  |
| 663 | CSP | 0.23 µM | BLI |  |
|  | (NANP)_5_ | 0.15 µM | SPR |  |

**Table S2:** BLI determined rate constants (*k*_a_, *k*_d_) and equilibrium dissociation constant (*K*_D_) obtained on blinded samples by the performing labs. BLI affinity against NANPx6 and FL-CSP targets were conducted on purified Fab fragments (Structural Biology Lab, US Military HIV Research Program, WRAIR) and BLI against FL-CSP target was conducted using intact IgG (Duke Human Vaccine Institute).

| **FL-CSP target - Fab binding kinetics** | | | | | | | | | | | | | |
| --- | --- | --- | --- | --- | --- | --- | --- | --- | --- | --- | --- | --- | --- |
| Fabs | | | *K*_D_ (M) | | *k*_a_ (1/Ms) | | *k*_d_ (1/s) | | Chi2 | | | R2 | |
| MGG4 | | | 5E-08 | | 5E+04 | | 2E-03 | | 3E-01 | | | 0.9998 | |
| cis43 | | | 2E-09 | | 3E+04 | | 5E-05 | | 3E-01 | | | 0.9998 | |
| 311 | | | 2E-10 | | 1E+05 | | 2E-05 | | 5E-02 | | | 0.9996 | |
| 317 | | | 2E-12 | | 1E+05 | | 3E-07 | | 9E-02 | | | 0.9994 | |
| 580 | | | 5E-07 | | 2E+05 | | 1E-01 | | 3E-03 | | | 0.998 | |
| 663 | | | 1E-07 | | 1E+05 | | 2E-02 | | 1E-01 | | | 0.9997 | |
| **NANPx6 peptide target - Fab binding kinetics** | | | | | | | | | | | | | |
| Fab | | | *K*_D_ (M) | | *k*_a_ (1/Ms) | | *k*_d_ (1/s) | | Chi2 | | | R2 | |
| MGG4 | | | 5E-08 | | 9E+04 | | 5E-03 | | 8E-02 | | | 0.999 | |
| cis43 | | | 4E-08 | | 4E+04 | | 2E-03 | | 1E-01 | | | 0.999 | |
| 311 | | | 7E-09 | | 3E+04 | | 2E-04 | | 4E-01 | | | 0.999 | |
| 317 | | | 3E-12 | | 6E+04 | | 2E-07 | | 4E-01 | | | 0.999 | |
| 580 | | | 3E-06 | | 1E+05 | | 4E-01 | | 6E-04 | | | 0.997 | |
| 663 | | | 1E-07 | | 4E+05 | | 6E-02 | | 3E-02 | | | 0.999 | |
| **FL-CSP target - Bivalent mAb binding kinetics** | | | | | | | | | | | | | |
| ***k*_a_ (1/Ms)** | *k*_a_ 1 | | *k*_a_ 2 | | *k*_a_ 3 | | *k*_a_ 4 | | Mean *k*_a_ | STD | | %CV | |
| mAb 663 | 9.66E+05 | | 8.87E+05 | | 1.06E+06 | | 9.49E+05 | | 9.65E+05 | 7.11E+04 | | 7.4 | |
| mAb 580 | 1.91E+06 | | 1.98E+06 | | 2.17E+06 | | 2.16E+06 | | 2.05E+06 | 1.31E+05 | | 6.4 | |
| mAb 311 | 4.51E+05 | | 3.74E+05 | | 5.15E+05 | | 5.89E+05 | | 4.82E+05 | 9.16E+04 | | 19.0 | |
| mAb 317 | 1.04E+06 | | 1.04E+06 | | 1.23E+06 | | 1.26E+06 | | 1.14E+06 | 1.15E+05 | | 10.1 | |
| mAb MGG4 | 4.60E+05 | | 4.81E+05 | | 4.63E+05 | | 4.92E+05 | | 4.74E+05 | 1.55E+04 | | 3.3 | |
| mAb CIS43 | 1.38E+06 | | 1.36E+06 | | 1.66E+06 | | 1.60E+06 | | 1.50E+06 | 1.51E+05 | | 10.1 | |
| mAb 1210 | 2.93E+05 | | 2.98E+05 | | 2.91E+05 | | 3.14E+05 | | 2.99E+05 | 1.03E+04 | | 3.4 | |
| ***k*_d_ (1/s)** | *k*_d_ 1 | | *k*_d_ 2 | | *k*_d_ 3 | | *k*_d_ 4 | | Mean *k*_d_ | STD | | %CV | |
| mAb 663 | 1.13E-03 | | 1.08E-03 | | 1.12E-03 | | 1.13E-03 | | 1.12E-03 | 2.39E-05 | | 2.1 | |
| mAb 580 | 1.18E-02 | | 1.33E-02 | | 1.30E-02 | | 1.37E-02 | | 1.29E-02 | 8.19E-04 | | 6.3 | |
| mAb 311 | 2.78E-05 | | 3.68E-05 | | 3.05E-05 | | 2.80E-05 | | 3.07E-05 | 4.20E-06 | | 13.7 | |
| mAb 317 | 3.97E-05 | | 3.04E-05 | | 2.30E-05 | | 2.56E-05 | | 2.97E-05 | 7.35E-06 | | 24.8 | |
| mAb MGG4 | 7.63E-05 | | 7.96E-05 | | 9.60E-05 | | 7.46E-05 | | 8.16E-05 | 9.83E-06 | | 12.0 | |
| mAb CIS43 | 6.37E-04 | | 6.66E-04 | | 7.21E-04 | | 7.44E-04 | | 6.92E-04 | 4.94E-05 | | 7.1 | |
| mAb 1210 | 2.65E-04 | | 2.63E-04 | | 1.91E-04 | | 2.68E-04 | | 2.47E-04 | 3.71E-05 | | 15.0 | |
| ***K*_D_ (M)** | *K*_D_ 1 | | *K*_D_ 2 | | *K*_D_ 3 | | *K*_D_ 4 | | Mean *K*_D_ | STD | | %CV | |
| mAb 663 | 1.17E-09 | | 1.22E-09 | | 1.06E-09 | | 1.20E-09 | | 1.16E-09 | 7.17E-11 | | 6.2 | |
| mAb 580 | 6.18E-09 | | 6.73E-09 | | 6.00E-09 | | 6.33E-09 | | 6.31E-09 | 3.13E-10 | | 5.0 | |
| mAb 311 | 6.15E-11 | | 9.82E-11 | | 5.91E-11 | | 4.75E-11 | | 6.66E-11 | 2.19E-11 | | 33.0 | |
| mAb 317 | 3.80E-11 | | 2.92E-11 | | 1.88E-11 | | 2.03E-11 | | 2.66E-11 | 8.90E-12 | | 33.5 | |
| mAb MGG4 | 1.66E-10 | | 1.66E-10 | | 2.08E-10 | | 1.52E-10 | | 1.73E-10 | 2.42E-11 | | 14.0 | |
| mAb CIS43 | 4.61E-10 | | 4.89E-10 | | 4.34E-10 | | 4.65E-10 | | 4.63E-10 | 2.25E-11 | | 4.9 | |

**Table S3:** Pearson correlation coefficient (r) and p values for *in vitro* and *in vivo* data from 3 independent challenge studies (yellow highlighted cells). Pearson's correlation coefficient |r| >0.7 suggested a strong linear association; |r| =0.4-0.7 suggested moderate linear association; and no linear association was inferred for |r| <0.4. Red r indicate strong associations and blue indicate p<0.05.

**Table S4:** ILSDA, Least Square (LS) mean (%) estimates and standard error obtained from a General Linear Model. ILSDA inhibitions from 3 or more independent experiments were used for this estimation.

| **mAb type** | **dose** | **Estimate** | **Standard Error** |
| --- | --- | --- | --- |
| **Negative** | **0.05** | 0.16 | 0.01 |
|  | **0.25** | 0.17 | 0 |
|  | **0.5** | 0.23 | 0.07 |
| **mAb311** | **0.05** | 0.23 | 0.08 |
|  | **0.25** | 0.38 | 0.09 |
|  | **0.5** | 0.72 | 0.08 |
| **mAb317** | **0.05** | 0.28 | 0.06 |
|  | **0.25** | 0.76 | 0.01 |
|  | **0.5** | 0.94 | 0.02 |
| **mAb580** | **0.05** | 0.31 | 0.07 |
|  | **0.25** | 0.54 | 0.03 |
|  | **0.5** | 0.65 | 0.05 |
| **mAb663** | **0.05** | 0.37 | 0.11 |
|  | **0.25** | 0.55 | 0.12 |
|  | **0.5** | 0.83 | 0.02 |
| **mAbCIS43** | **0.05** | 0.62 | 0.06 |
|  | **0.25** | 0.92 | 0.03 |
|  | **0.5** | 0.99 | 0 |

**Table S5:** ILSDA, Least Square mean differences between CSP mAbs at various dose levels and the negative control mAb 3G1. Standard error and p values are shown.

| Dose | mAb | Difference | Standard Error | p value |
| --- | --- | --- | --- | --- |
| 0.05 | 311 | 0.07 | 0.08 | 0.42 |
|  | 317 | 0.12 | 0.06 | 0.06 |
|  | 580 | 0.15 | 0.07 | 0.04 |
|  | 663 | 0.21 | 0.11 | 0.05 |
|  | CIS43 | 0.46 | 0.06 | <.0001 |
|  | MGG4 | 0.34 | 0.06 | <.0001 |
| 0.25 | 311 | 0.40 | 0.13 | 0.00 |
|  | 317 | 0.67 | 0.05 | <.0001 |
|  | 580 | 0.43 | 0.06 | <.0001 |
|  | 663 | 0.46 | 0.10 | <.0001 |
|  | CIS43 | 0.77 | 0.02 | <.0001 |
|  | MGG4 | 0.48 | 0.02 | <.0001 |
| 0.5 | 311 | 0.54 | 0.12 | <.0001 |
|  | 317 | 0.72 | 0.08 | <.0001 |
|  | 580 | 0.53 | 0.11 | <.0001 |
|  | 663 | 0.64 | 0.08 | <.0001 |
|  | CIS43 | 0.76 | 0.07 | <.0001 |
|  | MGG4 | 0.58 | 0.10 | <.0001 |

**Table S6:** ILSDA, pair-wise comparison of Least Square mean inhibition (%). Standard error and p values shown for selected groups of mAbs by dose level.

| Dose µg/ml | Reference | mAb | Estimate | Standard Error | p |
| --- | --- | --- | --- | --- | --- |
| 0.05 | MGG4 | 580 | 0.19 | 0.09 | 0.04 |
|  |  | 317 | 0.22 | 0.08 | 0.01 |
|  |  | 311 | 0.27 | 0.10 | 0.01 |
|  | CIS43 | 663 | 0.25 | 0.12 | 0.04 |
|  |  | 580 | 0.31 | 0.09 | 0.00 |
|  |  | 317 | 0.34 | 0.09 | 0.00 |
|  |  | 311 | 0.40 | 0.10 | 0.00 |
| 0.25 | 317 | 580 | -0.24 | 0.07 | 0.00 |
|  |  | MGG4 | -0.19 | 0.05 | 0.00 |
|  | CIS43 | MGG4 | -0.29 | 0.03 | <.0001 |
|  |  | 580 | 0.34 | 0.06 | <.0001 |
|  |  | 663 | 0.31 | 0.10 | 0.00 |
|  |  | 311 | 0.37 | 0.13 | 0.00 |
|  |  | 317 | 0.10 | 0.05 | 0.05 |
| 0.5 | 317 | 580 | -0.19 | 0.09 | 0.03 |
|  |  | 663 | -0.08 | 0.04 | 0.05 |
|  |  | MGG4 | -0.15 | 0.07 | 0.03 |
|  | CIS43 | MGG4 | -0.19 | 0.06 | 0.00 |
|  |  | 311 | 0.22 | 0.09 | 0.02 |
|  |  | 317 | 0.04 | 0.02 | 0.04 |
|  |  | 580 | 0.23 | 0.08 | 0.01 |
|  |  | 663 | 0.13 | 0.04 | 0.00 |

**Table S7:** ILSDA, estimated mAb dose level needed to achieve 80% inhibition, standard error and 95% confidence intervals are shown.

| **Response Level** | **mAb type** | **Estimate** | **Std. Error** | **95% CI** | | Test Difference against Reference (p values) | |  |  |  |  |  |
| --- | --- | --- | --- | --- | --- | --- | --- | --- | --- | --- | --- | --- |
|  |  | **µg/ml** |  |  |  |  |  |  |  |  |  |  |
| 80% | mAbCIS43 | 0.037 | 0.151 | -0.26 | 0.334 | Ref |  |  |  |  |  |  |
|  | mAb317 | 0.187 | 0.146 | -0.1 | 0.473 | 0.238 | Ref |  |  |  |  |  |
|  | mAb663 | 0.785 | 0.338 | 0.121 | 1.45 | 0.022 | 0.052 |  |  |  |  |  |
|  | mAb311 | 1.532 | 0.4 | 0.746 | 2.318 | 0.000 | 0.00 |  |  |  |  |  |
|  | mAb580 | 1.812 | 0.643 | 0.546 | 3.078 | 0.004 | 0.01 |  |  |  |  |  |
|  | mAb MGG4 | 2.448 | 1.524 | -0.549 | 5.445 | 0.058 | 0.07 |  |  |  |  |  |
|  | Negative | 11.485 | 101.238 | -187.64 | 210.613 | 0.455 | 0.46 |  |  |  |  |  |
|  | | | |  |  |  |  |  |  |  |  |  |
| *****There were no significant finding for paired comparisons with mAb663,  mAb311, mAb580, mAb MGG4 and negative control. | | | | | | | | |  |  |  |  |

**Table S8:** Pair-wise comparison p values of day 14 sterile protection frequency between vaccine groups using Fischer’s Exact Test (Left panel). Log-rank Test p values comparing survival curves (Right panel) Values <0.05 in red.

**Table S9: Raw data used for Statistical Analyses - Page 1**

|  | Protection (%) | | | | |
| --- | --- | --- | --- | --- | --- |
| mAb | Expt 1 24 hr (%) | Expt 1 48 hr (%) | Expt 2 24 hr (%) | Expt 3 24 hr | |
| 311 | 60% | 80% | 30% |  |  |
| 317 | 100% | 80% | 90% | 50% (15 ug) | 80% (50 ug) |
| 580 | 0% | 0% |  |  |  |
| 663 | 60% | 20% |  |  |  |
| CIS43 | 40% | 40% | 50% | 60% (15 ug) | 50% (50 ug) |
| MGG4 | 0% | 60% | 50% |  |  |
| Controls | 0% | 0% | 0% | 0% | |
|  |  |  |  |  |  |
|  | CSP OD=1 ELISA (ng/ml) | | | |  |
| mAb | Expt 1 | Expt 2 | Expt 3 | Expt 4 |  |
| 311 | 6 | 6 | 8 | 4 |  |
| 317 | 7 | 5 | 6 | 4 |  |
| 580 | 2 | 2 | 3 | 2 |  |
| 663 | 2 | 2 | 2 | 1 |  |
| CIS43 | 3 | 2 | 3 | 2 |  |
| MGG4 | 3 | 7 | 9 | 3 |  |
|  |  |  |  |  |  |
|  | SPZ ELISA OD=1 (ng/ml) | |  |  |  |
| mAb | Expt 1 | Expt 2 |  |  |  |
| 311 | 18 | 49 |  |  |  |
| 317 | 19 | 32 |  |  |  |
| 580 | 1905 | 1000 |  |  |  |
| 663 | 187 | 82 |  |  |  |
| CIS43 | 642 | 214 |  |  |  |
| MGG4 | 85 | 61 |  |  |  |
|  |  |  |  |  |  |
|  | NANPx6 ELISA OD=1 (ng/ml) | | | |  |
| mAb | Expt 1 | Expt 2 | Expt 3 | Expt 4 |  |
| 311 | 5 | 5 | 3 | 4 |  |
| 317 | 7 | 9 | 7 | 6 |  |
| 580 | 112 | 177 | 74 | 119 |  |
| 663 | 6 | 7 | 2 | 3 |  |
| CIS43 | 10 | 13 | 8 | 11 |  |
| MGG4 | 6 | 8 | 3 | 2 |  |

**Table S9: Raw data - Page 2**

| **mAb** | **ILSDA 5 ug/ml** | |  |  |  |
| --- | --- | --- | --- | --- | --- |
| 311 | 100% | 100% |  |  |  |
| 317 | 100% | 99% |  |  |  |
| 580 | 98% | 98% |  |  |  |
| 663 | 71% | 100% |  |  |  |
| CIS43 | 100% | 100% |  |  |  |
| MGG4 | 100% |  |  |  |  |
|  |  |  |  |  |  |
|  |  |  |  |  |  |
| **mAb** | **ILSDA 0.5 ug/ml** | | | | |
| 311 | 77% | 98% | 77% | 62% | 47% |
| 317 | 94% | 100% | 93% | 95% | 87% |
| 580 | 54% | 56% | 84% | 72% | 59% |
| 663 | 81% | 71% | 85% | 85% | 88% |
| CIS43 | 99% | 98% | 99% | 99% | 100% |
| MGG4 | 76% | 61% |  | 65% | 80% |
| 3G1 |  |  |  | 7% | 12% |
|  |  |  |  |  |  |
| **mAb** | **ILSDA 0.25 ug/ml** | | |  |  |
| 311 | 58% | 39% | 18% |  |  |
| 317 | 75% | 78% | 76% |  |  |
| 580 | 48% | 62% | 52% |  |  |
| 663 | 53% | 31% | 81% |  |  |
| CIS43 | 92% | 85% | 99% |  |  |
| MGG4 | 65% | 67% | 70% |  |  |
| 3G1 |  | 15% | 17% |  |  |
|  |  |  |  |  |  |
| **mAb** | **ILSDA 0.05 ug/ml** | | | | |
| 311 | 17% | 13% | -6% | -3% | 1% |
| 317 | 16% | -1% | 31% | 40% | 16% |
| 580 | 6% | 25% | 50% | 42% | 17% |
| 663 | 43% | 5% | 64% | 10% | 50% |
| CIS43 | 83% | 54% | 51% | 49% | 75% |
| MGG4 | 66% | 36% |  | 53% | 44% |
| 3G1 |  |  |  | -17% | 15% |

**Table S9: Raw data - Page 3**

|  | Avidity Index FL-CSP target | | | |
| --- | --- | --- | --- | --- |
| mAb | Expt 1 | Expt 2 | Expt 3 | Expt 4 |
| 311 | 52% | 71% | 71% | 62% |
| 317 | 81% | 93% | 102% | 100% |
| 580 | 4% | 17% | 15% | 5% |
| 663 | 65% | 72% | 62% | 88% |
| CIS43 | 30% | 32% | 24% | 36% |
| MGG4 | 72% | 55% | 42% | 74% |
|  |  |  |  |  |
|  | Avidity Index NANPx6 target | | | |
| mAb | Expt 1 | Expt 2 | Expt 3 | Expt 4 |
| 311 | 27% | 62% | 72% | 33% |
| 317 | 99% | 97% | 100% | 101% |
| 580 | 0% | 14% | 6% | 0% |
| 663 | 8% | 27% | 16% | 10% |
| CIS43 | 0% | 12% | 15% | 5% |
| MGG4 | 26% | 51% | 39% | 27% |
|  |  |  |  |  |
| mAb | **CSP Avidity (M NaSCN Dilution)** | | |  |
|  | Expt 1 | Expt 2 | Expt 3 |  |
| 311 | 3.08 | 3.42 | 3.3 |  |
| 317 | 4.35 | 4.65 | 4.3 |  |
| 580 | 2.31 | 2.67 | 2.5 |  |
| 663 | 3.74 | 3.9 | 3.8 |  |
| CIS43 | 3.06 | 4.18 | 3.4 |  |
| MGG4 | 3.24 | 3.59 | 3.3 |  |
|  |  |  |  |  |
|  | **NANP Avidity (M NaSCN Dilution)** | | |  |
| mAb | Expt 1 | Expt 2 | Expt 3 |  |
| 311 | 2.76 | 2.78 | 2.89 |  |
| 317 | 4.68 | 4.34 | 4.72 |  |
| 580 | 0.89 | 1.07 | 1.13 |  |
| 663 | 1.42 | 2.54 | 2.7 |  |
| CIS43 | 1.02 | 1.15 | 1.05 |  |
| MGG4 | 2.68 | 2.79 | 2.86 |  |

**Table S9: Raw data - Page 4**

|  | **Bivalent KD FL-CSP target** | | | |
| --- | --- | --- | --- | --- |
| mAb | Expt 1 | Expt 2 | Expt 3 | Expt 4 |
| 311 | 6.15E-11 | 9.82E-11 | 5.91E-11 | 4.75E-11 |
| 317 | 3.80E-11 | 2.92E-11 | 1.88E-11 | 2.03E-11 |
| 580 | 6.18E-09 | 6.73E-09 | 6.00E-09 | 6.33E-09 |
| 663 | 1.17E-09 | 1.22E-09 | 1.06E-09 | 1.20E-09 |
| CIS43 | 4.61E-10 | 4.89E-10 | 4.34E-10 | 4.65E-10 |
| MGG4 | 1.66E-10 | 1.66E-10 | 2.08E-10 | 1.52E-10 |
|  |  |  |  |  |
|  | Fab KD | |  |  |
| mAb | FL-CSP target | NANPx6 target |  |  |
| 311 | 2.01E-10 | 7.20E-09 |  |  |
| 317 | 2.20E-12 | 2.80E-12 |  |  |
| 580 | 5.24E-07 | 2.65E-06 |  |  |
| 663 | 1.39E-07 | 1.38E-07 |  |  |
| CIS43 | 1.70E-09 | 4.14E-08 |  |  |
| MGG4 | 4.74E-08 | 5.05E-08 |  |  |
|  |  |  |  |  |
|  | Buried Surface Area (BSA) Angstrom^2^ | |  |  |
| mAb | (NANP)n peptide | Junctional peptide |  |  |
| 311 | 540 | 540 |  |  |
| 317 | 574 | 574 |  |  |
| 580 | 351 | 351 |  |  |
| 663 | 403 | 403 |  |  |
| CIS43 | 484 | 624 |  |  |
| MGG4 | 385 | 385 |  |  |
